# Community Pharmacy Density Across Remote, Rural, Regional and Metropolitan Areas of Australia: An Ecological Study

**DOI:** 10.1111/ajr.70169

**Published:** 2026-03-25

**Authors:** Michael James Leach, Emily Griffin

**Affiliations:** ^1^ School of Rural Health Monash University Bendigo Victoria Australia

**Keywords:** accessibility, geographic information systems, health services research, medicines, pharmacy, subnational analysis

## Abstract

**Objective:**

To investigate community pharmacy density (CPD) at national and subnational (i.e., below‐national) levels in Australia, and examine area‐level factors associated with CPD.

**Methods:**

Aggregate, 2024 data on Australia's community pharmacies (CPs) and Statistical Area Level 3 (SA3) populations were sourced. CPD (number of CPs/10 000 population) was calculated nationally and sub‐nationally. SA3 characteristics associated with CPD quintiles were examined via partial proportional odds regression. Statistical significance was set at *p* < 0.05.

**Results:**

There were 6 032 CPs across 336 SA3s. CPD was 2.22, 2.09, and 2.63 CPs/10 000 population for Australia overall, metropolitan Australia, and remote/rural/regional Australia, respectively. SA3‐level data revealed CPD tended to be lower for outer‐ than inner‐city metropolitan areas. Clusters of SA3s with lowest‐quintile CPD (i.e., ‘pharmacy deserts’) were observed. Four large, remote SA3s in the Northern Territory (NT) (e.g., Daly‐Tiwi‐West Arnhem) had lowest‐quintile CPD, as did many outer‐city (e.g., Manningham East, Victoria) or other remote/rural/regional (e.g., Baw Baw, Victoria) SA3s. SA3 characteristics associated with a one‐quintile increase in CPD included remote/rural/regional (relative to metropolitan) areas (adjusted odds ratio [aOR] = 4.68, 95% confidence interval [CI] = 2.24–9.81) and percentage of male residents (aOR = 1.38, 95% CI = 1.16–1.63). Additionally, CPD tended to significantly increase with increasing percentages of residents aged 85+ or 65–84 years, and to significantly decrease with increasing SA3 area (km^2^).

**Conclusions:**

Correlates of CPD, and particular SA3s with lowest‐quintile CPD, could inform legislation, policies, and decisions related to CP premises and workforce Australia‐wide (e.g., Australia's Pharmacy Location Rules and state/territory‐level CP ownership legislation). Future studies of CPD across Australia should assess the correlation between CPD and general practice density, and incorporate additional subnational area characteristics (e.g., socioeconomic status) and CP accommodations (e.g., opening hours).

## Summary

- What is already known?
  ○ The International Pharmaceutical Federation reported that, in 2021, Australia's national‐level community pharmacy density was 2.26 community pharmacies per 10 000 population—below the global mean of 2.75 community pharmacies per 10 000 population.
  ○ Community pharmacy density has been investigated across subnational (i.e., below‐national) geographic areas in some countries—including Brazil, China, Malaysia, New Zealand, South Africa and United States—but not in Australia.
  ○ Characteristics of subnational areas associated with community pharmacy density have been investigated in Canada and New Zealand but not in Australia.
- What this paper adds?
  ○ In Australia during 2024, community pharmacy density was 2.22, 2.09 and 2.63 community pharmacies per 10 000 population for the whole country, metropolitan areas, and remote/rural/regional areas, respectively, consistent with literature suggesting that—despite having fewer pharmacists per community pharmacy—non‐metropolitan Australia is more reliant on community pharmacies due to a relative lack of other health services.
  ○ In Australia during 2024, characteristics of subnational geographic areas (i.e., Statistical Area Level 3 areas) independently associated with higher community pharmacy density included higher percentage of 85+‐year‐old or 65–84‐year‐old residents, higher percentage of male residents, and classification as remote/rural/regional, while lower community pharmacy density tended to be associated with higher area (km^2^)—information that could inform future research as well as legislation, policies, and decisions regarding community pharmacy and pharmacist distribution across Australia.
  ○ Choropleth mapping and tabulated area‐level data revealed that community pharmacy density was higher in inner‐metropolitan than outer‐metropolitan areas, that there were clusters of areas with very low (i.e., lowest‐quintile) community pharmacy density across certain parts of Australia (e.g., most of the Northern Territory, including Daly‐Tiwi‐West Arnhem), and that subnational areas across all states/territories of Australia had very low community pharmacy density (e.g., Manningham East and Baw Baw in Victoria)—further information pertinent to future research as well as legislation, policies, and decisions regarding community pharmacy and pharmacist distribution across Australia.

## 1 Introduction

Community pharmacies (CPs) provide specialist pharmaceutical and other health services to local communities and, as evidenced by the COVID‐19 pandemic, are essential [1, 2, 3]. The core services provided by CPs include dispensing and supplying prescription and non‐prescription medications, providing health advice for common ailments, and educating customers on medication use and safety. CPs typically offer further health services beyond these core services. In Australian CPs, there are five broad categories of services: clinical interventions aimed at minimising drug‐related problems, medication reviews (e.g., home medicines reviews), health promotion (e.g., vaccination), screening and disease state management for certain chronic diseases (e.g., diabetes), and addiction support services (e.g., opioid substitution) [2].

The geographical accessibility of CPs—and the services they offer—can be investigated using a range of indicators [4]. One such indicator is CP density (CPD). CPD measures the availability of CPs to people residing in a given geographic area [4]. It is typically expressed as the number of CPs per unit population (e.g., per 10 000 population) [4] or, less commonly, the ratio of CPs to resident population [5].

Internationally, CPD has mainly been calculated nationwide for certain countries. Among 77 countries in 2021, the mean national‐level CPD was 2.75 CPs/10 000 population [6]. The number of CPs/10 000 population in 2021 varied within and between the world's regions, ranging from 0.11 (Sudan) to 6.84 (Egypt) in Africa, 0.43 (Singapore) to 6.82 (Mongolia) in Asia, 0.88 (Denmark) to 5.82 (Malta) in Europe, 2.66 (United States [US]) to 3.06 (Costa Rica) in North America, 3.14 (Argentina) to 5.35 (Paraguay) in South America, and 0.81 (Fiji) to 2.26 (Australia) in Oceania [6]. Individual research studies have also reported national‐level CPD for particular countries [4, 7, 8, 9, 10, 11, 12, 13, 14, 15].

While national‐level CPD is important for purposes such as international benchmarking, subnational‐level (i.e., below‐national‐level) CPD is also required. In Australia, for example, subnational areas can be defined in a number of ways. A common approach to defining Australia's subnational areas is using hierarchical levels within the Main Structure of the Australian Statistical Geography Standard developed by the Australian Bureau of Statistics (ABS). These subnational levels are—in order of decreasing size—state/territory, Statistical Area (SA) Level 4 (SA4), SA Level 3 (SA3), SA Level 2 (SA2), SA Level 1 (SA1), and mesh blocks [16]. Investigating subnational CPD can reveal particular smaller geographic areas with relatively low and high numbers of CPs per unit population, thereby providing data to inform pharmaceutical health service policy and planning. Subnational CPD has been reported (via tables [5, 8, 10] and maps [8, 11, 12, 14, 17]) for particular countries other than Australia, including Brazil [17], China [14], Malaysia [5], New Zealand (NZ) [8], South Africa [10], and the US [11, 12]. Studies set in Brazil, China, NZ, and the US presented choropleth maps of CPD by subnational areas, thereby revealing clusters of geographic areas with relatively low numbers of CPs per unit population (termed ‘pharmacy deserts’ [8, 11, 12, 14, 17]).

Access to CPs is essential for all population subgroups across all populated geographic areas; however, the medical need for these services may be greater in some population subgroups (e.g., older adults) than others (e.g., younger adults) and, thus, greater in certain geographic areas than others [13]. Few studies have employed an ecological design to investigate characteristics of subnational areas associated with subnational‐level CPD [8, 13]. Sub‐nationally in Canada during 2019, non‐rurality (relative to rurality), lower household incomes, and higher population shares aged 65+ years were independently associated with significantly greater CPD. This Canadian study also found that, in most provinces, CPD was significantly lower or higher than the province of Ontario [13]. Sub‐nationally in NZ during 2020, relative to subnational areas classified as districts, subnational areas classified as cities were independently associated with significantly greater CPD [8]. Neither percentage of residents aged 65+ years, deprivation score, nor island of NZ were independently associated with CPD [8]. These results suggest, firstly, that CPD tends to be lower outside cities in Canada and NZ and, secondly, that CPD has been favourably influenced by markers of medical need in Canada but not NZ [8, 13]. Such results provide evidence to support the need for more equitable, needs‐based distribution of CPs in these countries.

No known studies have investigated subnational CPD in Australia, or examined characteristics of Australian subnational areas associated with CPD. Therefore, we aimed to firstly investigate CPD at national and subnational levels in Australia, and secondly to examine area‐level factors associated with CPD.

## 2 Methods

### 2.1 Study Design

We undertook an ecological study involving aggregate, area‐ and population‐level data. An ecological study design is a type of epidemiological study design whereby the data and the analysis are at the area and population level rather than the person level.

### 2.2 Setting

This study was set during 2024 in Australia at the national and subnational, or more specifically SA3, levels. SA3s have been chosen as the subnational areas for the present study because they are medium‐sized functional geographic areas—areas within which residents travel to access services such as CPs. Each SA3 is a region comprising multiple SA2 areas with similar characteristics. Most SA3s have populations of 30 000–130 000 people [18]. SA3s were used in the present study because reasonable travel distances/times to CPs would likely be overestimated or underestimated to greater extents by larger or smaller subnational areas than SA3s, respectively.

### 2.3 Data Sources

Data on all CP locations Australia‐wide were sourced from Geoscience Australia (access date: 23/5/2025) [19]. Data on Australia's population in mid‐2024 by age, sex and SA3s were sourced from the ABS via Data Explorer (access date: 1/10/2025) [20]. Data on Australia's 2021 SA3 boundaries and area (km^2^) for each SA3 were sourced from Esri (access date: 26/5/2025) [21]. Regarding remoteness classification, data on Australia's 2019 Modified Monash Model (MMM) boundaries and scores were sourced from the Australian Government Department of Health, Disability and Ageing (access date: 12/3/2025) [22].

### 2.4 Eligibility Criteria

Data for 336 of Australia's 340 spatial SA3s were included [18]. In line with past research [23], data for four very remote islands (i.e., Christmas Island, Cocos Island, Lord Howe Island and Norfolk Island) were excluded from our study. Data for non‐spatial SA3s (e.g., no usual address) were excluded because these lack exact locations [18].

### 2.5 Community Pharmacy Density

The outcome of interest was CPD: the number of CPs/10 000 population in each SA3. CPD was calculated at the area level for each SA3 separately. The calculation of CPD involved dividing the count of CPs in each SA3 by the corresponding population count, before multiplying by 10 000. This led to a continuous CPD variable. To assess whether this continuous variable would be a suitable outcome for linear regression, the normality of CPD was assessed via a Shapiro–Wilk test and histogram inspection. As CPD was non‐normally distributed (*p* < 0.001; figure not shown), the continuous CPD variable was collapsed into an ordinal categorical variable with five categories (i.e., quintiles)—a variable for use as the outcome in ordinal logistic regression (OLR).

Australia's nationwide CPD was also calculated by summing counts of CPs across all SA3s, summing population counts across all SA3s, dividing the sum of CP counts by the sum of population counts, and multiplying by 10 000. This calculation was also performed for all remote/rural/regional SA3s combined and all metropolitan SA3s combined.

### 2.6 Characteristics of Subnational Areas

Eight key demographic or geographic characteristics of SA3s were assessed. Continuous variables included percentage of residents aged < 65 years, percentage of residents aged 65–84 years, percentage of residents aged 85+ years, percentage of female residents, and percentage of male residents. Categorical variables included state/territory, area (km^2^) quintiles and remoteness classification based on MMM scores. State/territory was defined as two different polytomous variables. Firstly, for descriptive purposes, state/territory was classified using one category for each of Australia's eight states/territories. Secondly, for descriptive and regression analyses, five state/territory categories were used. Area (km^2^) quintiles were created from area (km^2^), forming a polytomous (ordinal) variable. Remoteness classification was defined as a polytomous (ordinal) variable and a binary variable. Firstly, for descriptive purposes, remoteness classification was categorised using one category for each MMM score. Secondly, for descriptive and regression analysis, remoteness classification was categorised using a binary variable with categories of remote/rural/regional (MMM scores 2–7; non‐reference group) and metropolitan (MMM score 1; reference group) [24]. It was necessary to collapse categories of state/territory and remoteness classification for regression analysis to obtain adequate cell sizes. Regarding remoteness, in instances where there were multiple MMM categories within one SA3, the MMM category with the highest (i.e., most metropolitan) MMM category was assigned. This approach ensured that any SA3 that was at least partly metropolitan was categorised as a metropolitan area, in line with the fact that metropolitan populations are typically larger than remote/rural/regional populations.

### 2.7 Community Pharmacy Mapping

The point layer of CP locations was mapped with overlaid service areas. The radius of the service areas was set at 25 km because, firstly, a Euclidean distance within 25 km is considered a reasonable travel distance to a CP [9], secondly, past NZ studies have used 25km‐radius pharmacy service areas [8, 9], and, thirdly, smaller service areas would be less ‘viewer‐friendly’ on a map of Australia—one of the world's largest countries.

A choropleth map of CPD quintiles across all SA3s Australia‐wide was produced. To provide greater clarity, a separate choropleth map was produced for the state/territory with the highest number of SA3s per square kilometre of area: Victoria [20, 21]. All CPD mapping was undertaken using ArcGIS Pro (Esri, Redlands, CA, USA). We have supplemented this map with a detailed table sorted in alphabetical order of SA3 in each state/territory (Appendix S1).

### 2.8 Data Analysis

SA3‐level analysis, with SA3s as the unit of analysis, was undertaken. Continuous and categorical variables were summarised using the median (interquartile range [IQR]) and frequencies (percentages), respectively. The continuous CPD variable was also summarised using the five‐number summary (minimum, lower quartile [Q1], median, upper quartile [Q3] and maximum) in a boxplot. Outlying CPD values were defined as values more than 1.5 times the IQR above Q3 or below Q1. Strengths of correlations between continuous CPD and each of the continuous SA3 characteristics was assessed using Pearson's correlation coefficient (*r*). Including both sex variables and all three age variables as exposures in a multivariable regression model would introduce multicollinearity issues because, for example, the percentage of female residents and the percentage of male residents are perfectly inversely correlated with one another (*r* = ±1). Therefore, only one of the two sex variables (i.e., percentage of male residents) and two of the three age group variables (i.e., percentage of 65–84‐year‐old residents and percentage of 85+‐year‐old residents) were included in regression modelling, alongside the remaining three SA3 characteristics. Univariable and multivariable proportional odds (PO) OLR models were initially used to assess associations between six SA3 characteristics (the exposures) and CPD quintiles (the outcome). The Wald test for parallel lines was used to assess the PO assumption for each SA3 characteristic [25]. As three SA3 characteristics (percentage of 65–84‐year‐old residents, percentage of 85+‐year‐old residents, and area [km^2^] quintiles) violated the PO assumption (*p* < 0.05), it was necessary to use partial PO OLR. While the beta coefficients in PO regression are the same (i.e., constrained) across all outcome categories, the effect estimates in *partial* PO regression are permitted to vary (i.e., unconstrained) across outcome categories [25]. Univariable PO regression models with constrained effect estimates were used for all SA3 characteristics except percentage of 65–84‐year‐old residents, percentage of 85+‐year‐old residents, and area (km^2^) quintiles, while a univariable partial PO regression model with unconstrained effect estimates was used for each of percentage of 65–84‐year‐old residents, percentage of 85+‐year‐old residents, and area (km^2^) quintiles. All six SA3 characteristics were included in a multivariable partial PO model, with unconstrained effect estimates for percentage of 65–84‐year‐old residents, percentage of 85+‐year‐old residents, and area (km^2^) quintiles, and constrained effect estimates for all other exposures. Univariable and multivariable OLR modelling involved computing unadjusted and adjusted odds ratios (ORs), respectively, alongside 95% confidence intervals (CIs) and *p*‐values. A *p* < 0.05 denoted statistical significance. Data analysis was undertaken in Stata v15.0 (StataCorp, College Station, TX, USA).

### 2.9 Ethical Considerations

Ethics approval was obtained from the Monash University Human Research Ethics Committee (project ID: 46001).

## 3 Results

**FIGURE 1:**
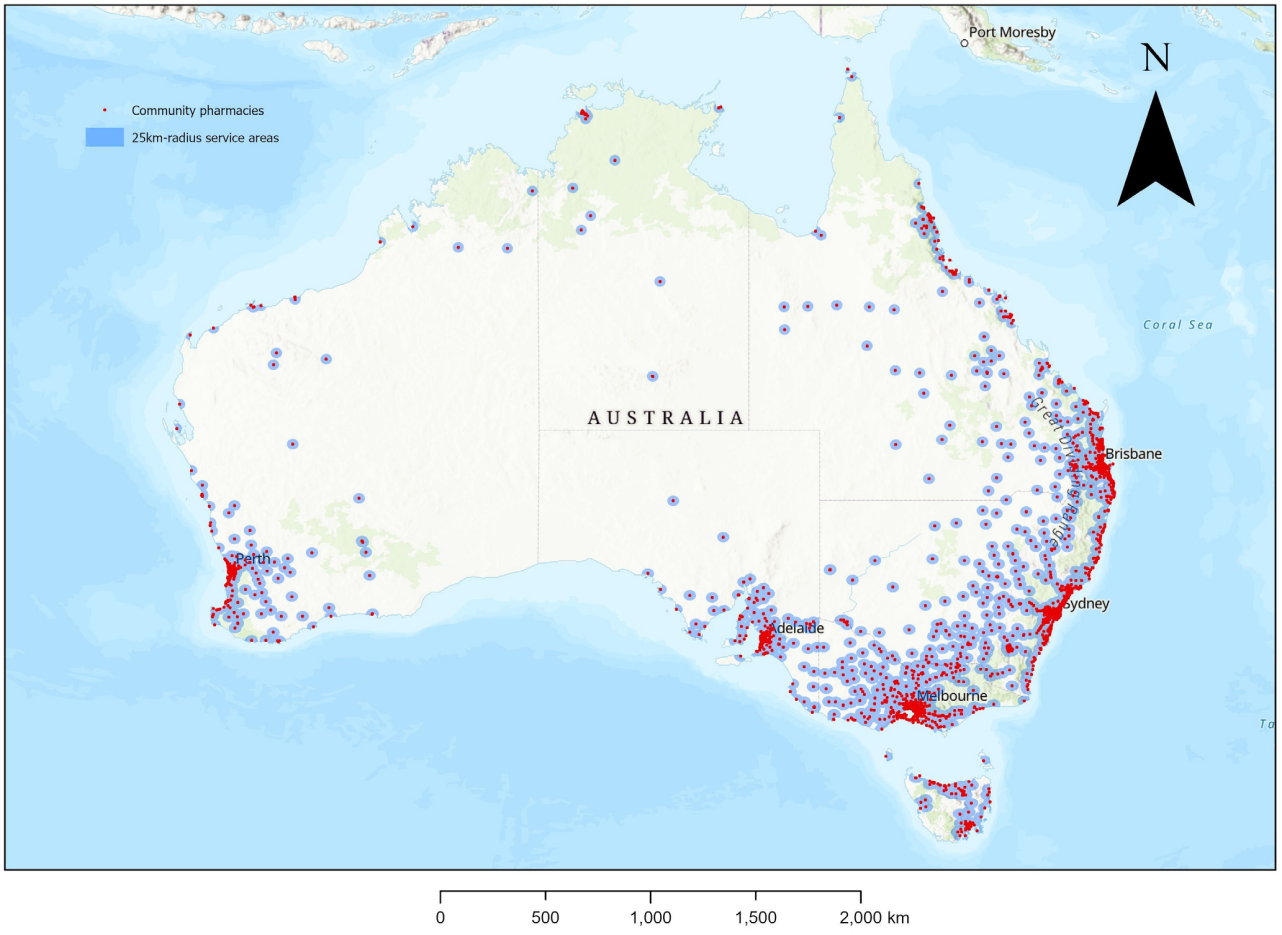
Community pharmacies and 25km‐radius community pharmacy service areas, Australia, 2024 (*N* = 6 032 community pharmacies). Abbreviation: km, kilometre.

In 2024, Australia had 6 032 CPs across 336 SA3s. Descriptive statistics on SA3s are shown in Table 1, while locations of CPs are shown in Figure 1. Large parts of Australia were not covered by 25 km‐radius service areas, particularly inland WA, NT, SA, Queensland and NSW. The relatively small state of Victoria had a relatively high proportionate coverage with 25km‐radius CP service areas. Nationally, Australia's CPD in 2024 was 2.22 CPs/10 000 population. CPD was 2.09 CPs/10 000 population across all metropolitan SA3s, and 2.63 CPs/10 000 population across all remote/rural/regional SA3s.

**TABLE 1:** Characteristics of Statistical Area Level 3 areas, overall and by quintiles of community pharmacy density (number of pharmacies per 10 000 population), Australia, 2024 (*N* = 336).

SA3 characteristics	Pharmacy density^a^—*n* (col. %)^b^	Pharmacy density^a^—*n* (row %)^b^					*M* (IQR)^b^
	Q1–Q5 [0–8.59], *N* = 336	Q1 [0–1.73], *N* = 68	Q2 [1.73–2.09], *N* = 67	Q3 [2.09–2.42], *N* = 67	Q4 [2.42–2.88], *N* = 67	Q5 [2.88–8.59], *N* = 67	Pharmacy density^a^
Age < 65 years (%)	*M* (IQR) = 81.82 (7.82)	*M* (IQR) = 85.98 (7.42)	*M* (IQR) = 83.11 (6.35)	*M* (IQR) = 81.17 (7.00)	*M* (IQR) = 80.09 (8.89)	*M* (IQR) = 78.65 (8.15)	*r* = −0.30
Age 65–84 years (%)	*M* (IQR) = 15.93 (7.08)	*M* (IQR) = 12.43 (6.27)	*M* (IQR) = 14.72 (5.14)	*M* (IQR) = 16.48 (6.51)	*M* (IQR) = 17.19 (8.17)	*M* (IQR) = 18.89 (7.85)	*r* = 0.28
Age 85+ years (%)	*M* (IQR) = 2.32 (1.17)	*M* (IQR) = 1.43 (1.15)	*M* (IQR) = 2.07 (0.96)	*M* (IQR) = 2.53 (1.08)	*M* (IQR) = 2.61 (0.80)	*M* (IQR) = 2.51 (0.89)	*r* = 0.35
Female (%)	*M* (IQR) = 50.38 (1.53)	*M* (IQR) = 50.33 (1.20)	*M* (IQR) = 50.47 (1.22)	*M* (IQR) = 50.68 (1.12)	*M* (IQR) = 50.64 (1.52)	*M* (IQR) = 49.38 (1.78)	*r* = −0.22
Male (%)	*M* (IQR) = 49.62 (1.53)	*M* (IQR) = 49.67 (1.20)	*M* (IQR) = 49.53 (1.22)	*M* (IQR) = 49.32 (1.12)	*M* (IQR) = 49.36 (1.52)	*M* (IQR) = 50.62 (1.78)	*r* = 0.22
State (separate)							
NSW	92 (27.4%)	12 (13.0%)	19 (20.7%)	18 (19.6%)	29 (31.5%)	14 (15.2%)	2.40 (0.77)
QLD	82 (24.4%)	24 (29.3%)	18 (22.0%)	11 (13.4%)	12 (14.6%)	17 (20.7%)	2.08 (0.96)
VIC	66 (19.6%)	13 (19.7%)	16 (24.2%)	14 (21.2%)	12 (18.2%)	11 (16.7%)	2.21 (0.79)
WA	34 (10.1%)	6 (17.6%)	5 (14.7%)	11 (32.4%)	3 (8.8%)	9 (26.5%)	2.19 (1.02)
SA	28 (8.3%)	1 (3.6%)	4 (14.3%)	8 (28.6%)	7 (25.0%)	8 (28.6%)	2.49 (1.01)
TAS	15 (4.5%)	2 (13.3%)	3 (20.0%)	2 (13.3%)	2 (13.3%)	6 (40.0%)	2.76 (1.64)
ACT	10 (3.0%)	6 (60.0%)	1 (10.0%)	1 (10.0%)	0 (0%)	2 (20.0%)	1.59 (0.98)
NT	9 (2.7%)	4 (44.4%)	1 (11.1%)	2 (22.2%)	2 (22.2%)	0 (0%)	2.05 (0.97)
State/territory (collapsed)							
NSW + ACT	102 (30.4%)	18 (17.6%)	20 (19.6%)	19 (18.6%)	29 (28.4%)	16 (15.7%)	2.37 (0.92)
QLD	82 (24.4%)	24 (29.3%)	18 (22.0%)	11 (13.4%)	12 (14.6%)	17 (20.7%)	2.08 (0.96)
VIC	66 (19.6%)	13 (19.7%)	16 (24.2%)	14 (21.2%)	12 (18.2%)	11 (16.7%)	2.21 (0.79)
WA + TAS + NT	58 (17.3%)	12 (20.7%)	9 (15.5%)	15 (25.9%)	7 (12.1%)	15 (25.9%)	2.19 (1.02)
SA	28 (8.3%)	1 (3.6%)	4 (14.3%)	8 (28.6%)	7 (25.0%)	8 (28.6%)	2.49 (1.01)
Area (km^2^) quintile							
Q1 [10.7–41.2]	68 (20.2%)	13 (19.1%)	10 (14.7%)	10 (14.7%)	19 (27.9%)	16 (23.5%)	2.45 (0.90)
Q2 [42.4–129.6]	67 (19.9%)	16 (23.9%)	18 (26.9%)	20 (29.9%)	7 (10.4%)	6 (9.0%)	2.04 (0.65)
Q3 [132.4–918.9]	67 (19.9%)	22 (32.8%)	23 (34.3%)	14 (20.9%)	7 (10.4%)	1 (1.5%)	1.97 (0.55)
Q4 [929.7–8 434.0]	67 (19.9%)	11 (16.4%)	10 (14.9%)	15 (22.4%)	19 (28.4%)	11 (17.9%)	2.34 (0.75)
Q5 [8 566.8–714 555.6]	67 (19.9%)	6 (9.0%)	6 (9.0%)	8 (11.9%)	15 (22.4%)	32 (47.8%)	2.87 (1.20)
Remoteness (separate)							
1 (Metropolitan areas)	202 (60.1%)	54 (26.7%)	51 (25.2%)	44 (21.8%)	31 (15.3%)	22 (10.9%)	2.06 (0.77)
2 (Regional centres)	47 (14.0%)	6 (12.8%)	9 (19.1%)	12 (25.5%)	12 (25.5%)	8 (17.0%)	2.30 (0.77)
3 (Large rural towns)	42 (12.5%)	1 (2.4%)	5 (11.9%)	7 (16.7%)	15 (35.7%)	14 (33.3%)	2.69 (0.69)
4 (Medium rural towns)	25 (7.4%)	1 (4.0%)	1 (4.0%)	2 (8.0%)	8 (32.0%)	13 (52.0%)	2.88 (0.84)
5 (Small rural towns)	8 (2.4%)	2 (25.0%)	0 (0%)	0 (0%)	0 (0%)	6 (75.0%)	4.16 (2.20)
6 (Remote communities)	8 (2.4%)	2 (25.0%)	1 (12.5%)	2 (25.0%)	1 (12.5%)	2 (25.0%)	2.24 (1.38)
7 (Very remote communities)	4 (1.2%)	2 (50.0%)	0 (0%)	0 (0%)	0 (0%)	2 (50.0%)	2.72 (3.23)
Remoteness classification (collapsed)							
1 (Metropolitan areas)	202 (60.1%)	54 (26.7%)	51 (25.2%)	44 (21.8%)	31 (15.3%)	22 (10.9%)	2.06 (0.77)
2–7 (Remote/rural/regional areas)	134 (39.9%)	14 (10.4%)	16 (11.9%)	23 (17.2%)	36 (26.9%)	45 (33.6%)	2.64 (0.83)
All SA3s (*N* = 336)	M (IQR) = 2.22 (0.91)	68 (20.2%)	67 (19.9%)	67 (19.9%)	67 (19.9%)	67 (19.9%)	—

Abbreviations: ACT, Australian Capital Territory; col., column; disp., dispensing; IQR, interquartile range; M, median; *n*, frequency; *N*, sample size; NSW, New South Wales; NT, Northern Territory; Q1, lowest quintile; Q2, lower quintile; Q3, middle quintile; Q4, higher quintile; Q5, highest quintile; QLD, Queensland; *r*, Pearson's correlation coefficient; SA, South Australia; SA3, Statistical Area Level 3; TAS, Tasmania; WA, Western Australia.

^a^ Number of community pharmacies per 10 000 population in each Statistical Area Level 3 area.

^b^ Unless otherwise stated.

**FIGURE 2:**
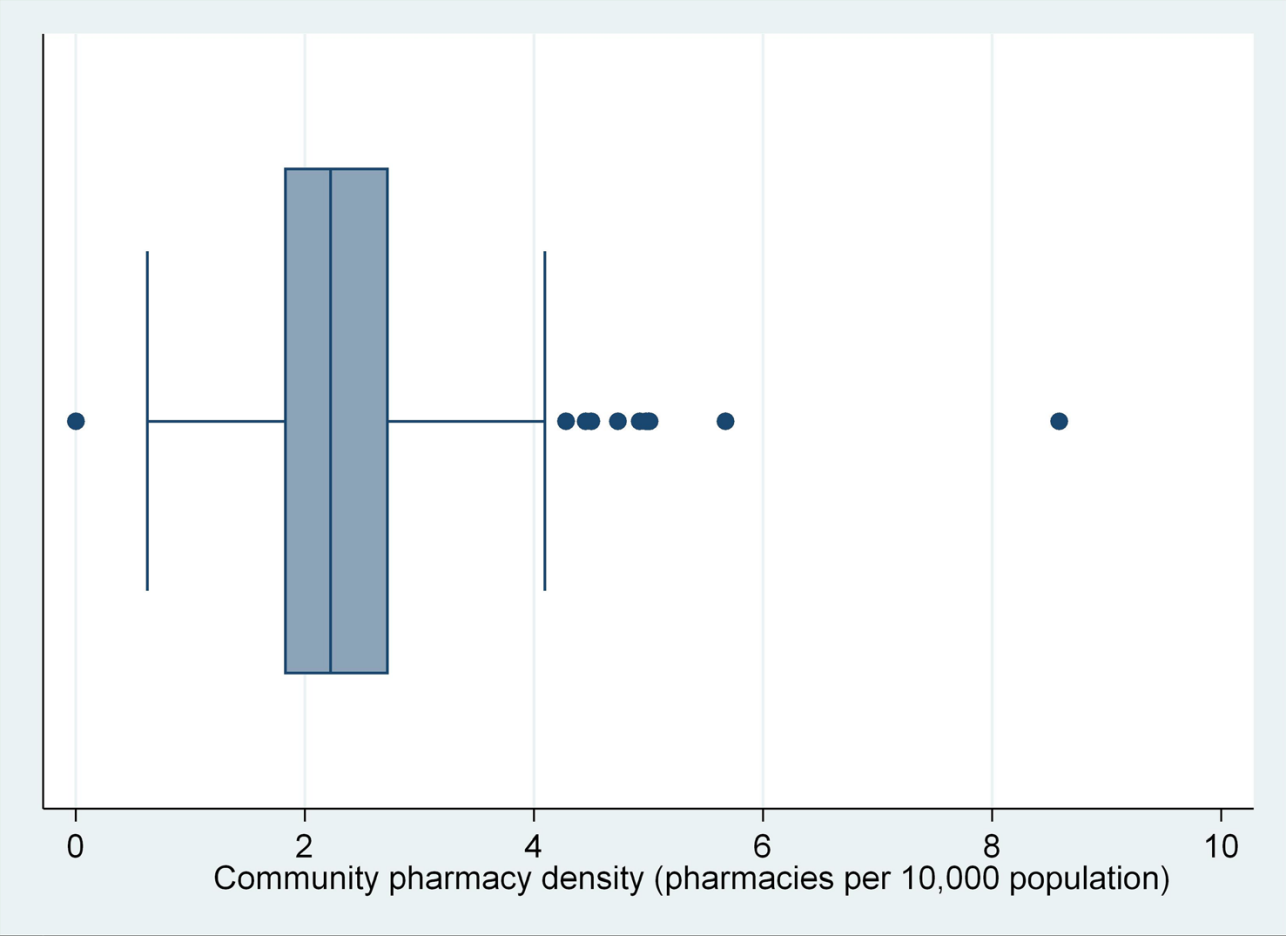
Boxplot showing community pharmacy density (number of pharmacies per 10 000 population) in each Statistical Area Level 3 area, Australia, 2024 (*N* = 336). Abbreviation: pop., population.

Sub‐nationally, the minimum, Q1, median, Q3 and maximum CPD values were 0, 1.82, 2.22, 2.73 and 8.59 CPs/10 000 population in each SA3, respectively (Figure 2). There were 15 outliers: 10 with very high CPDs of ≥ 4.10 CPs/10 000 population, and 5 with very low CPDs of zero (Appendix S1). The five SA3s with no CPs were Uriarra‐Namadgi in ACT, Blue Mountains‐South, Illawarra Catchment Reserve, and Jervis Bay in NSW, and Daly‐Tiwi‐West Arnhem in NT. Four of these five SA3s had small populations ≤ 624, while the very remote community of Daly‐Tiwi‐West Arnhem had a much larger population of 18 374.

**FIGURE 3:**
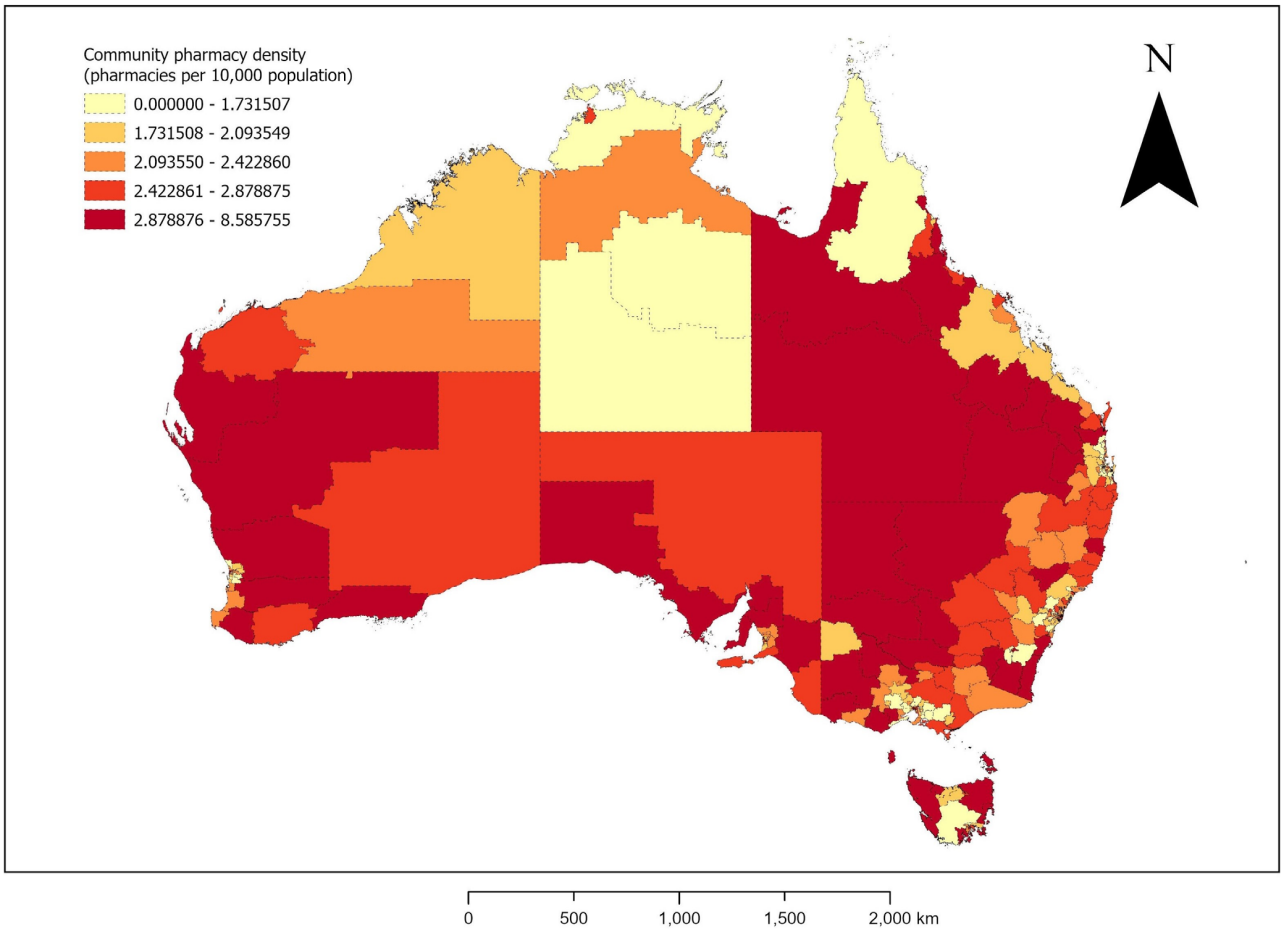
Quintiles of community pharmacy density (number of pharmacies per 10 000 population) by Statistical Area Level 3 areas, Australia, 2024. Abbreviations: km, kilometre; pop., population.

**FIGURE 4:**
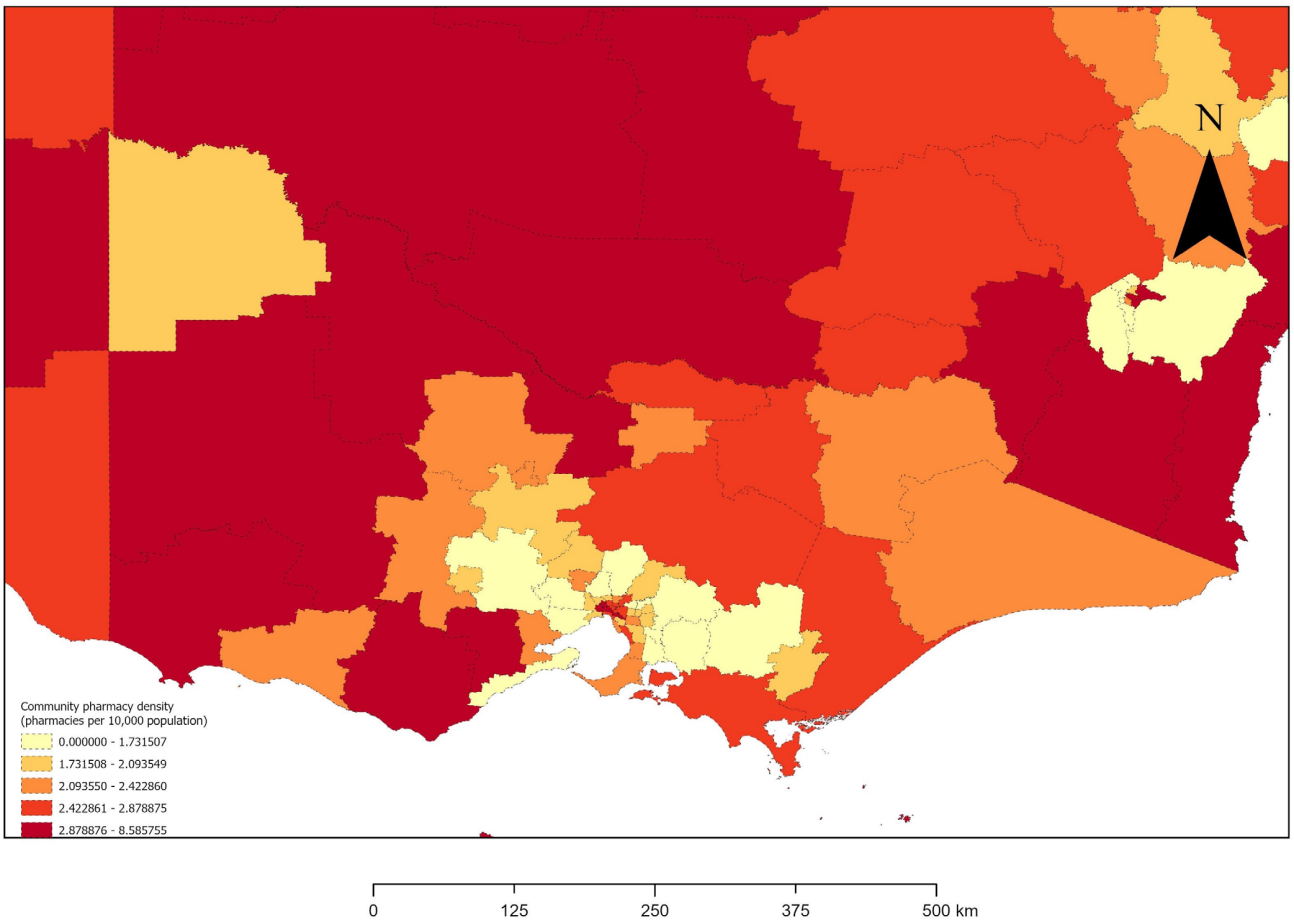
Quintiles of community pharmacy density (pharmacies per 10 000 population) by Statistical Area Level 3 areas, Victoria, 2024. Abbreviations: km, kilometre; pop., population.

Continuous CPD (number of CPs per 10 000 population) was collapsed into quintiles: lowest (quintile 1 [0–1.73]), lower (quintile 2 [1.73–2.09]), middle (quintile 3 [2.09–2.42]), higher (quintile 4 [2.42–2.88]), and highest (quintile 5 [2.88–8.59]). Figures 3 and 4 are choropleth maps of CPD quintiles for Australia and Victoria, respectively. Appendix S1 shows CPD for each SA3 across all states/territories of Australia. Many SA3s with highest and higher CPD tended to be clustered together. In each of NSW and Queensland, there were geographically large (i.e., high area [km^2^]), inland, remote/rural/regional SA3s with highest quintile CPD. There were, however, 14 remote/rural/regional SA3s with lowest‐quintile CPD: four in NT (Alice Springs, Barkly, Daly‐Tiwi‐West Arnhem and East Arnhem), three in NSW (Blue Mountains‐South, Illawarra Catchment Reserve and Jervis Bay), two in Queensland (Cairns‐North and Far North), two in Tasmania (Brighton and Central Highlands), two in Victoria (Baw Baw and Creswick‐Daylesford‐Ballan), and one in ACT (Uriarra‐Namadgi). Of the Australian state and territory with no metropolitan SA3s, Tasmania had some areas with highest‐quintile CPD (e.g., the regional centre of Hobart Inner) while none of NT's SA3s had highest‐quintile CPD. In ACT, South and North Canberra had highest‐quintile and lower‐quintile CPD, respectively. Highest‐quintile CPD was observed for the inner metropolitan areas of NSW (Sydney Inner City), Queensland (Brisbane Inner), Victoria (Melbourne City), SA (Adelaide City) and WA (Perth City). All states/territories except SA and WA had at least one SA3 with lowest‐quintile CPD. Across each Australian state/territory except NT and Tasmania, most or all SA3s with lowest‐quintile CPD were metropolitan rather than remote/rural/regional SA3s. Those metropolitan SA3s with lowest‐quintile CPD tended to be outer‐city areas. In Victoria, for example, the 13 SA3s with lowest‐quintile CPD tended to be located on—or just beyond—the outskirts of metropolitan Melbourne: four to the west of inner Melbourne (Wyndham, Melton‐Bacchus Marsh, Surf Coast‐Bellarine Peninsula and Creswick‐Daylesford‐Ballan), two to the north of inner Melbourne (Tullamarine‐Broadmeadows and Whittlesea‐Wallan), and six to the east of inner Melbourne (Mannington‐West, Mannington‐East, Yarra Ranges, Casey‐North, Casey‐South, Cardinia and Baw Baw). Of these 13 Victorian SA3s with lowest‐quintile CPD, only two (Creswick‐Daylesford‐Ballan and Baw Baw) are remote/rural/regional areas.

Univariable (crude ORs [cORs]) and multivariable (adjusted ORs [aORs]) associations between SA3 characteristics and CPD quintiles are shown in Table 2. The adjusted odds of a one‐quintile increase in CPD were 4.68‐fold greater (aOR = 4.68, 95% CI = 2.24–9.81) for remote/rural/regional than metropolitan areas. For every 1% increase in the percentage of male residents, the adjusted odds of a one‐quintile increase in CPD were 38% greater (aOR = 1.38, 95% CI = 1.16–1.63). There were significant associations between percentage of the population aged 85+ years and CPD quintiles for three of the four comparisons between CPD quintiles, and a significant association between percentage of the population aged 65–84 years and CPD quintiles for the remaining one comparison between CPD quintiles. For example, relative to the lowest (quintile 1) CPD quintile, the adjusted odds of the lower (quintile 2), middle (quintile 3), higher (quintile 4), or highest (quintile 5) CPD quintiles were 10.24‐fold greater (aOR = 10.24, 95% CI = 4.50–23.26) for every 1% increase in the percentage of residents aged 85+ years. There were also significant associations between area (km^2^) quintiles and CPD quintiles for 10 comparisons, with CPD tending to decrease with increasing area (km^2^). For example, relative to the lowest, lower, middle, and higher CPD quintiles, the adjusted odds of the highest CPD quintile were 75% (aOR = 0.25, 95% CI = 0.09–0.72), 99% (aOR = 0.01, 95% CI = 0.001–0.13), 95% (aOR = 0.05, 95% CI = 0.01–0.19), and 78% (aOR = 0.22, 95% CI = 0.07–0.70) lower for the lower, middle, higher, and highest area (km^2^) quintiles, respectively, than the lowest area (km^2^) quintile.

**TABLE 2:** Associations between Statistical Area Level 3 characteristics and quintiles of community pharmacy density (number of pharmacies per 10 000 population), Australia, 2024 (*N* = 336).

SA3 characteristics	Proportional odds		Reference group(s) for unconstrained effects							
	Pharmacy density^a^ Q1–Q5 [0–8.59]		Pharmacy density^a^ Q1 [0–1.73]		≤ Pharmacy density^a^ Q2 [1.73–2.09]		≤ Pharmacy density^a^ Q3 [2.09–2.42]		≤ Pharmacy density^a^ Q4 [2.42–2.88]	
	cOR (95% CI)	aOR^b^ (95% CI)	cOR (95% CI)	aOR^b^ (95% CI)	cOR (95% CI)	aOR^b^ (95% CI)	cOR (95% CI)	aOR^b^ (95% CI)	cOR (95% CI)	aOR^b^ (95% CI)
Age 65–84 years (%)	—	—	1.18 (1.10–1.26)*	0.91 (0.79–1.04)	1.15 (1.10–1.21)*	0.97 (0.87–1.09)	1.13 (1.08–1.18)*	1.10 (0.99–1.22)	1.11 (1.05–1.16)*	1.13 (1.01–1.28)*
Age 85+ years (%)	—	—	4.14 (2.79–6.14)*	10.24 (4.50–23.26)*	2.80 (2.09–3.76)*	5.36 (2.70–10.66)*	2.10 (1.59–2.77)*	1.96 (1.01–3.79)*	1.69 (1.23–2.31)*	1.31 (0.63–2.73)
Male (%)	1.13 (1.02–1.26)*	1.38 (1.16–1.63)*	—	—	—	—	—	—	—	—
State/territory (collapsed)										
NSW + ACT	1.00	1.00	—	—	—	—	—	—	—	—
QLD	0.67 (0.40–1.14)	0.87 (0.49–1.56)	—	—	—	—	—	—	—	—
VIC	0.81 (0.47–1.39)	0.57 (0.31–1.05)	—	—	—	—	—	—	—	—
WA + TAS + NT	1.03 (0.58–1.83)	1.22 (0.63–2.34)	—	—	—	—	—	—	—	—
SA	1.89 (0.93–3.87)	1.25 (0.57–2.73)	—	—	—	—	—	—	—	—
Area (km^2^) quintiles										
Q1 [10.7–41.2]	—	—	1.00	1.00	1.00	1.00	1.00	1.00	1.00	1.00
Q2 [42.4–129.6]	—	—	0.75 (0.33–1.72)	1.21 (0.49–2.95)	0.50 (0.25–0.99)*	0.54 (0.26–1.15)	0.23 (0.11–0.49)*	0.18 (0.08–0.41)*	0.32 (0.12–0.88)*	0.25 (0.09–0.72)*
Q3 [132.4–918.9]	—	—	0.48 (0.22–1.07)	0.95 (0.36–2.52)	0.25 (0.12–0.51)*	0.21 (0.09–0.50)*	0.13 (0.05–0.31)*	0.05 (0.02–0.15)*	0.05 (0.01–0.38)*	0.01 (0.001–0.13)*
Q4 [929.7–8 434.0]	—	—	1.20 (0.50–2.92)	0.79 (0.22–2.90)	1.12 (0.54–2.30)	0.29 (0.09–0.89)*	0.81 (0.41–1.60)	0.08 (0.03–0.25)*	0.71 (0.31–1.64)	0.05 (0.01–0.19)*
Q5 [8 566.8–714 555.6]	—	—	2.40 (0.85–6.76)	1.40 (0.34–5.74)	2.34 (1.05–5.22)*	0.58 (0.17–1.91)	2.22 (1.09–4.49)*	0.22 (0.08–0.67)*	2.97 (1.42–6.21)*	0.22 (0.07–0.70)*
Remoteness (collapsed)										
1 (Metropolitan areas)	1.00	1.00	—	—	—	—	—	—	—	—
2–7 (Remote/rural/regional areas)	3.94 (2.61–5.95)*	4.68 (2.24–9.81)*	—	—	—	—	—	—	—	—

Abbreviations: ACT, Australian Capital Territory; aOR, adjusted odds ratio; CI, confidence interval; cOR, crude (unadjusted) odds ratio; MMM, Modified Monash Model; NSW, New South Wales; NT, Northern Territory; Q1, lowest quintile; Q2, lower quintile; Q3, middle quintile; Q4, higher quintile; Q5, highest quintile; QLD, Queensland; SA, South Australia; SA3, Statistical Area Level 3; TAS, Tasmania; WA, Western Australia.

^a^ Number of community pharmacies per 10 000 population in each Statistical Area Level 3 area.

^b^ Adjusted for all other variables in the multivariable partial proportional odds ordinal logistic regression model (i.e., all other Statistical Area Level 3 characteristics in this table).

* Statistically significant at the 5% level (*p* < 0.05).

## 4 Discussion

Australia‐wide in 2024, CPD was 2.22 CPs/10 000 population. This result is similar to Australia's CPD 3 years earlier: 2.26 CPs/10 000 population [6]. Australia's CPD in 2024 was approximately half a CP below the international mean of 2.75 CPs/10 000 population, despite Australia having very high human development [6, 26]. Another country in Oceania, NZ, had a similar CPD in 2020 to Australia in 2024, despite also having very high human development [8, 26]. There is scope to increase Australia's national‐level CPD up to or beyond the international mean of 2.75 CPs/10 000 population through evidence‐based and legislation‐based approaches.

Our study's significant, positive associations between percentages of oldest‐old (85+‐year‐old) and youngest‐old (65–84‐year‐old) residents and CPD are partially supported by Canadian research, which found a significant, positive association between share of 65+‐year‐old residents and CPD [13]. Our result suggests the establishment of CPs in Australia's SA3s may have responded—in part—to the medical needs of older residents. At the population level, the number of health conditions that people experience increases with increasing age. Australia‐wide during 2022, for example, 50% of people aged 65+ years lived with two or more chronic conditions while only 30% of people aged 45–64 years lived with two or more chronic conditions [27]. Our study's significant, positive association between percentage of male residents and CPD may be unrelated to medical needs among Australian men, who have proportionately fewer chronic conditions at the population level (47.5% in 2022) than Australian women (52.3% in 2022) [27]. As our ecological study was conducted at one time point only, it is unclear whether CPs were established in SA3 areas with more men or, alternatively, more men moved to SA3 areas where CPs had already been established. The association between sex and CPD has not been previously investigated in the literature and requires further investigation.

The significantly lower CPDs observed with increasing SA3 area (km^2^) suggest a need for more CPs in SA3s that are ≥ 42.4 km^2^. The tendency for CPD to decrease with increasing area (km^2^) may reflect the inherent difficulty of providing services to geographically dispersed populations. Associations between sizes of subnational areas and CPD have not previously been investigated in the literature and require further investigation. The states/territories of Australia's SA3s were found to be unrelated to CPD. While a past Canadian study found statistically significant variation in CPD across provinces [13], our study is the first to investigate variation in CPD across Australia's states/territories.

In our Australian study, CPD was significantly higher in remote/rural/regional than metropolitan SA3s. This result cannot be explained by the higher median area (km^2^) of remote/rural/regional (7 785 km^2^) compared with metropolitan (78 km^2^) SA3s (author calculations using data in Appendix S1) because area (km^2^) was one of five other SA3 characteristics controlled for in the multivariable regression analysis. The significantly higher CPD in remote/rural/regional than metropolitan SA3s is partly supported by a qualitative study in which medical practitioners, nurses, and allied health professionals perceived CPs and community pharmacists to be widely accessible in remote/rural/regional Australia [28]. This result does, however, contradict findings from past Canadian and NZ ecological studies, which found that CPD was significantly higher outside than inside cities [8, 13]. Australia may be doing more than some other countries to ensure CPs are available to people residing outside cities. For example, among the 23 Australian university campuses offering accredited pharmacy degrees, the percentages of campus locations in metropolitan and remote/rural/regional areas are not too dissimilar: 56.5% (13 of 23) and 43.5% (10 of 23) in metropolitan and remote/rural/regional areas, respectively (author calculations using [29]). Our mapped and tabulated SA3‐level data suggest that the significantly lower CPD in metropolitan SA3s is primarily due to lower CPD in outer‐city, rather than inner‐city, areas. While this finding has not been previously reported, the Australian Government has reported that Australia's outer‐metropolitan areas have lower access to general practitioners (GP) than Australia's inner‐metropolitan areas [30]. The significantly higher CPD in remote/rural/regional areas ostensibly suggests that Australia has prioritised remote/rural/regional people's needs over metropolitan people's needs. This is not necessarily the case, however, because the contexts of remote/rural/regional and metropolitan areas are different. In remote/rural/regional Australia, community pharmacists tend to take on a more generalised healthcare role and act as intermediaries between patients and other healthcare professionals (e.g., GPs). In remote/rural/regional communities without other health services, CPs are first points of contact for patients; remote/rural/regional communities tend to be more reliant on CPs than other health services [31, 32]. Moreover, a given remote/rural/regional CP may only have one pharmacist and, in 2019, there were fewer pharmacists per 100 000 population in remote/rural/regional than metropolitan areas of Australia [31, 33]. Therefore, while many remote/rural/regional areas of Australia have relatively high numbers of CPs per unit population, they may nevertheless experience a key workforce issue: too few community pharmacists per CP.

While there are subnational areas of Australia that do not appear to need any more CPs, there are also subnational areas of Australia that do appear to need more CPs. Our study is the first to tabulate or map CPD sub‐nationally in Australia. We found that populations in large parts of Australia were very well serviced by CPs, including but not limited to the inner‐city areas of Sydney, Brisbane, Melbourne, Adelaide, and Perth, as well as clusters of inland, remote/rural/regional SA3s in NSW and Queensland. SA3s with very low CPD (i.e., pharmacy deserts) were, however, evident across all Australian states/territories. Large parts of NT had very low CPD, as did outer‐city areas of ACT and outer‐city areas of all Australian states. Our results suggest that, in the capital cities of Australian states, CPs tended to be concentrated in inner‐city areas and relatively sparse in outer‐city areas. It is, thus, necessary to increase CPD across certain areas of remote/rural/regional and metropolitan Australia. In particular, it is necessary to prioritise establishment of CPs in SA3s that have no CPs (e.g., NT's remote, 18 374‐strong community of Daly‐Tiwi‐West Arnhem) or low CPD (e.g., Victoria's metropolitan area of Manningham‐East and rural town of Baw Baw), while simultaneously considering factors such as local medical needs, availability of CPs in neighbouring SA3s, and relevant legislation (e.g., Australia's Pharmacy Location Rules [34]).

Our study has limitations. Firstly, regarding remoteness, the MMM measure does not distinguish between Australia's inner‐metropolitan and outer‐metropolitan areas. Therefore, our descriptive and regression analysis results do not reflect the variation in CPD between inner‐ and outer‐city areas observed in our mapped and tabulated SA3‐level data. Secondly, as populations tend to be smaller in Australia's remote/rural/regional (median = 42 983; IQR = 36 499) than metropolitan (median = 90 086; IQR = 73 596) SA3s (author calculations using data from Appendix S1), remote/rural/regional SA3s tend to be more sensitive to small changes in pharmacy count. When slightly increasing the relatively small number of CPs in certain SA3s with relatively small population sizes (e.g., Esperance), the health system necessarily overcorrects and gives relatively high CPDs in such areas (Appendix S1). This overcorrection is not problematic: it is better for a particular geographic area to be somewhat over‐serviced than under‐serviced by CPs, unless there happens to be a nearby area that is in greater need of another CP. Thirdly, regression results could have been subject to confounding bias. Examples of unmeasured confounding factors that were unavailable in our data sources include area‐level socio‐economic status (SES), population health status (e.g., number of chronic diseases), and transport‐related factors (e.g., availability of public transport). Fourthly, our study did not consider other types of access beyond availability of CPs, such as accommodations (e.g., opening hours) [11, 35]. In particular, due to well‐established under‐resourcing [36], remote/rural/regional CPs may be unable to offer as many accommodations as metropolitan CPs.

Our study also has strengths. Firstly, there were no missing data; the variables assessed in the present study were 100% complete due to the use of data from well‐maintained, secondary data sources [19, 20, 21, 22]. Secondly, remoteness was systematically assessed using the same classification system that is employed by the Australian Government Department of Health, Disability and Ageing: the MMM [24]. Thirdly, unlike a past NZ study [9], our study produced a map of CP service areas that do not overlap with surrounding seas and, thus, do not overestimate the coverage of service areas.

## 5 Conclusions

This novel ecological study has presented data on CPD Australia‐wide and across subnational (i.e., SA3) areas of Australia during 2024, and identified characteristics of SA3s that are independently associated with CPD. Australia's nationwide CPD of 2.22 CPs/10 000 population is less than the international mean of 2.75 CPs/10 000 population [6]. CPD was significantly higher in SA3s with more older (65–84‐ or 85+‐year‐old) and male residents, and in remote/rural/regional SA3s than metropolitan SA3s. The latter result is partly attributable to the relatively low CPDs of outer‐metropolitan areas, and may reflect greater reliance on CPs in under‐resourced remote/rural/regional areas with few other health services. Additionally, CPD tended to significantly decrease as the area (km^2^) of SA3s increased. Future Australian studies in this area should incorporate CP accommodations, include additional factors in multivariable models of area‐level characteristics associated with CPD, and assess the correlation between CPD and general practice density. Additionally, in future, studies similar to our Australian study and an equivalent NZ study [8] could be conducted sub‐nationally in other countries. SA3 characteristics associated with CPD in our study suggest particular types of SA3s that should be prioritised in initiatives aimed at achieving a more equitable distribution of CPs and community pharmacists across Australia. We revealed subnational areas of Australia with highest, higher, middle, lower and lowest CPD quintiles through choropleth maps and a table, which could inform Australian decision‐makers and policy‐makers at local, state, and national levels. Subnational areas of Australia with zero or lowest‐quintile CPD (e.g., Daly‐Tiwi‐West Arnhem in NT, Manningham East in Victoria, and Baw Baw in Victoria) should be prioritised in legislation, policies, and decisions concerning the establishment of CPs throughout Australia and Australia's CP workforce. For example, our results can be used to inform updates to Australia's Pharmacy Location Rules [34] and state/territory‐level CP ownership legislation (e.g., Victoria's Pharmacy Regulation Act 2010 [37] and NT's Pharmacy Ownership Standard [38]).

## Author Contributions

**Michael James Leach:** conceptualisation (lead), data curation (lead), formal analysis (lead), investigation (lead), methodology (lead), project administration (lead), resources (lead), software (lead), validation (lead), visualisation (lead), writing – original draft preparation (lead), writing – review and editing (lead). **Emily Griffin:** conceptualisation (supporting), methodology (supporting), writing – review and editing (supporting).

## Funding

The authors have nothing to report.

## Disclosure

M.J.L.'s regular salary, which covers research, is paid by the Australian Government Department of Health, Disability and Ageing. The Australian Government Department of Health, Disability and Ageing was not involved in any aspect of the study design, data collection, data analysis, interpretation of results, writing of the article, or decision to submit the article for publication.

## Ethics Statement

Ethics approval to undertake this study was obtained from the Monash University Human Research Ethics Committee (project ID: 46001).

## Conflicts of Interest

The authors declare no conflicts of interest.

## Supporting information


**Appendix S1:** ajr70169‐sup‐0001‐AppendixS1.docx.

## Data Availability

The data that supports the findings of this study are available in the Supporting Information of this article.
