# Supplementary material for: Community Pharmacy Density Across Remote, Rural, Regional and Metropolitan Areas of Australia: An Ecological Study
Source: Aust J Rural Health. 2026 Mar 25;34(2):e70169. doi: 10.1111/ajr.70169 (PMC13014210; doi:10.1111/ajr.70169)
Supplement: Supplementary file 1 — Appendix S1: ajr70169‐sup‐0001‐AppendixS1.docx. [file AJR-34-0-s001.docx]

**Supplementary Appendix S1 Table A.1:** Statistical Area Level 3 (SA3) characteristics, estimated resident population, number of community pharmacies, community pharmacy density, and community pharmacy density quintiles for each SA3 area, sorted in alphabetical order of SA3 in each state/territory, Australia, 2024

| **State/territory** | **Statistical Area Level 3** | **MMM remoteness category** | **Area (km^2^)** | **% <65 years** | **% 65-84 years** | **% 85+ years** | **% female** | **% male** | **ERP** | **No. of CPs** | **CPD** | **CPD quintile** |
| --- | --- | --- | --- | --- | --- | --- | --- | --- | --- | --- | --- | --- |
| ACT | Belconnen | Metropolitan Areas | 112.15 | 84.89 | 13.35 | 1.76 | 50.56 | 49.44 | 108,483 | 17 | 1.57 | 1 |
| ACT | Canberra East^*^ | Metropolitan Areas | 211.92 | 84.91 | 14.24 | 0.85 | 32.9 | 67.1 | 2,009 | 1 | 4.98 | 5 |
| ACT | Gungahlin | Metropolitan Areas | 91.86 | 92.1 | 7.26 | 0.64 | 50.25 | 49.75 | 92,178 | 11 | 1.19 | 1 |
| ACT | Molonglo | Metropolitan Areas | 27.15 | 96.08 | 3.7 | 0.22 | 50.59 | 49.41 | 16,026 | 2 | 1.25 | 1 |
| ACT | North Canberra | Metropolitan Areas | 44.72 | 88.94 | 9.44 | 1.62 | 50.71 | 49.29 | 66,338 | 12 | 1.81 | 2 |
| ACT | South Canberra | Metropolitan Areas | 44.41 | 81.21 | 16.25 | 2.54 | 51.5 | 48.5 | 33,697 | 11 | 3.26 | 5 |
| ACT | Tuggeranong | Metropolitan Areas | 159.71 | 82.79 | 15.48 | 1.73 | 50.96 | 49.04 | 89,340 | 12 | 1.34 | 1 |
| ACT | Uriarra - Namadgi^*^ | Regional Centres | 1,619.51 | 89.9 | 9.94 | 0.16 | 46.79 | 53.21 | 624 | 0 | 0.00 | 1 |
| ACT | Weston Creek | Metropolitan Areas | 18.15 | 79.58 | 17.56 | 2.86 | 51.54 | 48.46 | 24,724 | 4 | 1.62 | 1 |
| ACT | Woden Valley | Metropolitan Areas | 28.6 | 82.38 | 14.57 | 3.05 | 51.72 | 48.28 | 40,436 | 9 | 2.23 | 3 |
| NSW | Albury | Regional Centres | 5,597.49 | 79.49 | 18.1 | 2.41 | 50.79 | 49.21 | 69,679 | 19 | 2.73 | 4 |
| NSW | Armidale | Large Rural Towns | 16,395.65 | 79.56 | 17.91 | 2.53 | 51.51 | 48.49 | 38,154 | 9 | 2.36 | 3 |
| NSW | Auburn | Metropolitan Areas | 33.06 | 89.69 | 9.24 | 1.07 | 46.62 | 53.38 | 113,554 | 23 | 2.03 | 2 |
| NSW | Bankstown | Metropolitan Areas | 68.62 | 84.91 | 13.01 | 2.08 | 50.22 | 49.78 | 193,256 | 46 | 2.38 | 3 |
| NSW | Bathurst | Large Rural Towns | 6,984.18 | 80.37 | 17.2 | 2.43 | 49.11 | 50.89 | 50,529 | 10 | 1.98 | 2 |
| NSW | Baulkham Hills | Metropolitan Areas | 72.03 | 81.82 | 15.76 | 2.42 | 50.79 | 49.21 | 159,856 | 27 | 1.69 | 1 |
| NSW | Blacktown | Metropolitan Areas | 56.31 | 85.75 | 12.67 | 1.58 | 49.47 | 50.53 | 147,185 | 34 | 2.31 | 3 |
| NSW | Blacktown - North | Metropolitan Areas | 103.07 | 92.6 | 6.79 | 0.61 | 49.62 | 50.38 | 176,444 | 11 | 0.62 | 1 |
| NSW | Blue Mountains | Metropolitan Areas | 942.41 | 76.66 | 21.04 | 2.3 | 51.3 | 48.7 | 78,604 | 14 | 1.78 | 2 |
| NSW | Blue Mountains – South^*^ | Small Rural Towns | 2,456.91 | 42.86 | 57.14 | 0 | 42.86 | 57.14 | 7 | 0 | 0.00 | 1 |
| NSW | Botany | Metropolitan Areas | 28.09 | 88.01 | 10.4 | 1.59 | 49.23 | 50.77 | 62,328 | 17 | 2.73 | 4 |
| NSW | Bourke - Cobar - Coonamble^*^ | Small Rural Towns | 162,766.16 | 79.77 | 17.76 | 2.47 | 49.65 | 50.35 | 22,005 | 11 | 5.00 | 5 |
| NSW | Bringelly - Green Valley | Metropolitan Areas | 291.47 | 90.37 | 8.75 | 0.88 | 50.18 | 49.82 | 170,887 | 19 | 1.11 | 1 |
| NSW | Broken Hill and Far West | Large Rural Towns | 146,860.78 | 76.61 | 20.37 | 3.02 | 50.76 | 49.24 | 19,840 | 8 | 4.03 | 5 |
| NSW | Camden | Metropolitan Areas | 73.87 | 87.89 | 10.81 | 1.3 | 50.88 | 49.12 | 77,749 | 14 | 1.80 | 2 |
| NSW | Campbelltown (NSW) | Metropolitan Areas | 334.22 | 86.43 | 12.36 | 1.21 | 50.54 | 49.46 | 189,943 | 33 | 1.74 | 2 |
| NSW | Canada Bay | Metropolitan Areas | 19.75 | 82.49 | 14.93 | 2.58 | 51.42 | 48.58 | 89,946 | 21 | 2.33 | 3 |
| NSW | Canterbury | Metropolitan Areas | 29.96 | 85.11 | 12.55 | 2.34 | 48.52 | 51.48 | 146,381 | 38 | 2.60 | 4 |
| NSW | Carlingford | Metropolitan Areas | 25.69 | 84.35 | 13.56 | 2.09 | 50.13 | 49.87 | 78,354 | 13 | 1.66 | 1 |
| NSW | Chatswood - Lane Cove | Metropolitan Areas | 33.17 | 85 | 12.77 | 2.23 | 51.36 | 48.64 | 124,750 | 32 | 2.57 | 4 |
| NSW | Clarence Valley | Large Rural Towns | 9,263.19 | 71.8 | 24.82 | 3.38 | 49.53 | 50.47 | 55,716 | 14 | 2.51 | 4 |
| NSW | Coffs Harbour | Large Rural Towns | 3,966.56 | 75.66 | 21.3 | 3.04 | 51.2 | 48.8 | 94,847 | 26 | 2.74 | 4 |
| NSW | Cronulla - Miranda - Caringbah | Metropolitan Areas | 55.6 | 79.77 | 16.92 | 3.31 | 50.89 | 49.11 | 123,132 | 33 | 2.68 | 4 |
| NSW | Dapto - Port Kembla | Metropolitan Areas | 163.7 | 80.83 | 16.47 | 2.7 | 50.35 | 49.65 | 82,396 | 15 | 1.82 | 2 |
| NSW | Dubbo | Large Rural Towns | 29,738.42 | 80.41 | 17.04 | 2.55 | 49.68 | 50.32 | 76,236 | 20 | 2.62 | 4 |
| NSW | Dural - Wisemans Ferry | Metropolitan Areas | 569.24 | 78.79 | 18.26 | 2.95 | 49.61 | 50.39 | 28,423 | 8 | 2.81 | 4 |
| NSW | Eastern Suburbs - North | Metropolitan Areas | 26.18 | 84.4 | 13.06 | 2.54 | 51.98 | 48.02 | 133,328 | 43 | 3.23 | 5 |
| NSW | Eastern Suburbs - South | Metropolitan Areas | 31.55 | 85.62 | 12.14 | 2.24 | 50.76 | 49.24 | 144,589 | 38 | 2.63 | 4 |
| NSW | Fairfield | Metropolitan Areas | 123.77 | 82.06 | 15.82 | 2.12 | 50.51 | 49.49 | 198,113 | 48 | 2.42 | 3 |
| NSW | Gosford | Metropolitan Areas | 988.41 | 76.6 | 20.23 | 3.17 | 51.37 | 48.63 | 181,658 | 40 | 2.20 | 3 |
| NSW | Goulburn - Mulwaree | Large Rural Towns | 9,099.91 | 77.85 | 19.51 | 2.64 | 49.18 | 50.82 | 39,562 | 9 | 2.27 | 3 |
| NSW | Great Lakes | Large Rural Towns | 3,133.05 | 63.34 | 32.13 | 4.53 | 51.01 | 48.99 | 33,811 | 9 | 2.66 | 4 |
| NSW | Griffith - Murrumbidgee (West) | Large Rural Towns | 28,859.72 | 81.78 | 15.62 | 2.6 | 49.31 | 50.69 | 49,946 | 16 | 3.20 | 5 |
| NSW | Hawkesbury | Metropolitan Areas | 2,492.01 | 82.39 | 16.15 | 1.46 | 48.86 | 51.14 | 25,243 | 3 | 1.19 | 1 |
| NSW | Hornsby | Metropolitan Areas | 137.4 | 83.92 | 13.97 | 2.11 | 50.67 | 49.33 | 90,755 | 19 | 2.09 | 2 |
| NSW | Hurstville | Metropolitan Areas | 34.01 | 82.12 | 15.17 | 2.71 | 50.39 | 49.61 | 138,899 | 38 | 2.74 | 4 |
| NSW | Illawarra Catchment Reserve^*^ | Regional Centres | 744.34 | 100.00 | 0 | 0 | 80 | 20 | 5 | 0 | 0.00 | 1 |
| NSW | Inverell - Tenterfield | Medium Rural Towns | 30,568.64 | 72.31 | 24.51 | 3.18 | 50.07 | 49.93 | 39,310 | 10 | 2.54 | 4 |
| NSW | Jervis Bay^*^ | Medium Rural Towns | 67.81 | 82.57 | 15.46 | 1.97 | 50.66 | 49.34 | 304 | 0 | 0.00 | 1 |
| NSW | Kempsey - Nambucca | Medium Rural Towns | 5,339.33 | 71.44 | 25.6 | 2.96 | 49.78 | 50.22 | 52,761 | 18 | 3.41 | 5 |
| NSW | Kiama - Shellharbour | Metropolitan Areas | 390.8 | 79.07 | 18.5 | 2.43 | 50.87 | 49.13 | 104,623 | 22 | 2.10 | 3 |
| NSW | Kogarah - Rockdale | Metropolitan Areas | 31.35 | 84.46 | 13.09 | 2.45 | 49.87 | 50.13 | 155,571 | 41 | 2.64 | 4 |
| NSW | Ku-ring-gai | Metropolitan Areas | 85.55 | 80.78 | 15.9 | 3.32 | 51.54 | 48.46 | 128,989 | 31 | 2.40 | 3 |
| NSW | Lachlan Valley | Medium Rural Towns | 41,000.72 | 75.94 | 20.92 | 3.14 | 49.83 | 50.17 | 54,344 | 15 | 2.76 | 4 |
| NSW | Lake Macquarie - East | Metropolitan Areas | 138.09 | 78.27 | 18.8 | 2.93 | 50.8 | 49.2 | 129,234 | 27 | 2.09 | 2 |
| NSW | Lake Macquarie - West | Metropolitan Areas | 507.63 | 77.71 | 19.47 | 2.82 | 50.75 | 49.25 | 90,226 | 17 | 1.88 | 2 |
| NSW | Leichhardt | Metropolitan Areas | 10.66 | 85.25 | 13.13 | 1.62 | 51.82 | 48.18 | 58,273 | 15 | 2.57 | 4 |
| NSW | Lithgow - Mudgee | Medium Rural Towns | 16,044.63 | 76.77 | 20.66 | 2.57 | 49.2 | 50.8 | 48,327 | 13 | 2.69 | 4 |
| NSW | Liverpool | Metropolitan Areas | 123.83 | 87.86 | 10.75 | 1.39 | 49.85 | 50.15 | 147,623 | 26 | 1.76 | 2 |
| NSW | Lower Hunter | Metropolitan Areas | 8,566.78 | 82.82 | 15.55 | 1.63 | 48.98 | 51.02 | 105,038 | 20 | 1.90 | 2 |
| NSW | Lower Murray^*^ | Large Rural Towns | 63,968.37 | 77.4 | 20.07 | 2.53 | 48.23 | 51.77 | 13,332 | 6 | 4.50 | 5 |
| NSW | Maitland | Metropolitan Areas | 380.12 | 84.38 | 13.86 | 1.76 | 50.83 | 49.17 | 94,799 | 16 | 1.69 | 1 |
| NSW | Manly | Metropolitan Areas | 14.35 | 84.31 | 13.49 | 2.2 | 51.11 | 48.89 | 45,173 | 9 | 1.99 | 2 |
| NSW | Marrickville - Sydenham - Petersham | Metropolitan Areas | 12.67 | 87.69 | 10.53 | 1.78 | 49.97 | 50.03 | 57,207 | 13 | 2.27 | 3 |
| NSW | Merrylands - Guildford | Metropolitan Areas | 55.8 | 86.71 | 11.62 | 1.67 | 48.94 | 51.06 | 172,650 | 30 | 1.74 | 2 |
| NSW | Moree - Narrabri | Medium Rural Towns | 32,026.52 | 81.41 | 16.48 | 2.11 | 49.45 | 50.55 | 24,828 | 6 | 2.42 | 3 |
| NSW | Mount Druitt | Metropolitan Areas | 82.71 | 87.69 | 11.11 | 1.2 | 50.63 | 49.37 | 118,825 | 20 | 1.68 | 1 |
| NSW | Newcastle | Metropolitan Areas | 249.87 | 83 | 14.69 | 2.31 | 50.3 | 49.7 | 187,897 | 49 | 2.61 | 4 |
| NSW | North Sydney - Mosman | Metropolitan Areas | 18.98 | 81.25 | 16.17 | 2.58 | 53.03 | 46.97 | 99,529 | 32 | 3.22 | 5 |
| NSW | Orange | Large Rural Towns | 6,267.53 | 81.17 | 16.55 | 2.28 | 50.68 | 49.32 | 62,800 | 14 | 2.23 | 3 |
| NSW | Parramatta | Metropolitan Areas | 47.09 | 88.27 | 10.24 | 1.49 | 48.83 | 51.17 | 162,662 | 29 | 1.78 | 2 |
| NSW | Pennant Hills - Epping | Metropolitan Areas | 22.17 | 83.51 | 14.12 | 2.37 | 51.11 | 48.89 | 57,379 | 10 | 1.74 | 2 |
| NSW | Penrith | Metropolitan Areas | 356.86 | 86.68 | 11.93 | 1.39 | 50.5 | 49.5 | 169,142 | 36 | 2.13 | 3 |
| NSW | Pittwater | Metropolitan Areas | 90.65 | 76.86 | 20.03 | 3.11 | 50.87 | 49.13 | 64,366 | 14 | 2.18 | 3 |
| NSW | Port Macquarie | Large Rural Towns | 3,687.77 | 70.5 | 25.59 | 3.91 | 51.59 | 48.41 | 90,903 | 25 | 2.75 | 4 |
| NSW | Port Stephens | Metropolitan Areas | 1,071.98 | 71.56 | 25.34 | 3.1 | 50.21 | 49.79 | 79,356 | 19 | 2.39 | 3 |
| NSW | Queanbeyan | Metropolitan Areas | 6,511.19 | 86.27 | 12.42 | 1.31 | 49.12 | 50.88 | 68,473 | 9 | 1.31 | 1 |
| NSW | Richmond - Windsor | Metropolitan Areas | 178.84 | 83.27 | 14.41 | 2.32 | 50.03 | 49.97 | 39,434 | 10 | 2.54 | 4 |
| NSW | Richmond Valley - Coastal | Regional Centres | 1,572.89 | 76.01 | 21.12 | 2.87 | 51.45 | 48.55 | 91,379 | 26 | 2.85 | 4 |
| NSW | Richmond Valley - Hinterland | Large Rural Towns | 7,390.60 | 76.68 | 20.7 | 2.62 | 50.53 | 49.47 | 71,639 | 20 | 2.79 | 4 |
| NSW | Rouse Hill - McGraths Hill | Metropolitan Areas | 118.22 | 90.89 | 8.3 | 0.81 | 50.11 | 49.89 | 74,414 | 8 | 1.08 | 1 |
| NSW | Ryde - Hunters Hill | Metropolitan Areas | 47.16 | 84.5 | 12.93 | 2.57 | 50.64 | 49.36 | 158,204 | 34 | 2.15 | 3 |
| NSW | Shoalhaven | Large Rural Towns | 4,374.43 | 71.35 | 25.34 | 3.31 | 50.13 | 49.87 | 110,537 | 32 | 2.89 | 5 |
| NSW | Snowy Mountains | Medium Rural Towns | 14,283.34 | 79.19 | 18.37 | 2.44 | 48.1 | 51.9 | 21,296 | 7 | 3.29 | 5 |
| NSW | South Coast | Medium Rural Towns | 9,864.85 | 67.67 | 28.96 | 3.37 | 50.53 | 49.47 | 78,003 | 23 | 2.95 | 5 |
| NSW | Southern Highlands | Large Rural Towns | 2,330.02 | 71.77 | 24.75 | 3.48 | 51.43 | 48.57 | 53,517 | 14 | 2.62 | 4 |
| NSW | St Marys | Metropolitan Areas | 33.11 | 86.47 | 12.39 | 1.14 | 49.65 | 50.35 | 59,138 | 16 | 2.71 | 4 |
| NSW | Strathfield - Burwood - Ashfield | Metropolitan Areas | 34.14 | 86.14 | 11.47 | 2.39 | 49.88 | 50.12 | 172,236 | 37 | 2.15 | 3 |
| NSW | Sutherland - Menai - Heathcote | Metropolitan Areas | 240.25 | 82.02 | 15.68 | 2.3 | 50.75 | 49.25 | 114,213 | 23 | 2.01 | 2 |
| NSW | Sydney Inner City | Metropolitan Areas | 25.05 | 90.96 | 7.99 | 1.05 | 47.4 | 52.6 | 243,970 | 95 | 3.89 | 5 |
| NSW | Tamworth - Gunnedah | Large Rural Towns | 20,155.09 | 79.7 | 17.64 | 2.66 | 50.07 | 49.93 | 87,997 | 21 | 2.39 | 3 |
| NSW | Taree - Gloucester | Large Rural Towns | 6,675.07 | 69.12 | 27.4 | 3.48 | 50.69 | 49.31 | 58,837 | 16 | 2.72 | 4 |
| NSW | Tumut - Tumbarumba | Medium Rural Towns | 9,174.77 | 75.44 | 21.93 | 2.63 | 49.38 | 50.62 | 14,971 | 6 | 4.01 | 5 |
| NSW | Tweed Valley | Metropolitan Areas | 1,307.58 | 72.99 | 23.11 | 3.9 | 51.5 | 48.5 | 99,793 | 26 | 2.61 | 4 |
| NSW | Upper Hunter | Medium Rural Towns | 11,472.41 | 81.33 | 16.37 | 2.3 | 49.04 | 50.96 | 31,223 | 9 | 2.88 | 5 |
| NSW | Upper Murray exc. Albury | Large Rural Towns | 28,231.96 | 69.65 | 26.74 | 3.61 | 49.84 | 50.16 | 44,453 | 15 | 3.37 | 5 |
| NSW | Wagga Wagga | Large Rural Towns | 18,950.57 | 80.09 | 17.23 | 2.68 | 50.15 | 49.85 | 100,935 | 28 | 2.77 | 4 |
| NSW | Warringah | Metropolitan Areas | 149.21 | 83.1 | 14.02 | 2.88 | 50.75 | 49.25 | 161,233 | 33 | 2.05 | 2 |
| NSW | Wollondilly | Metropolitan Areas | 870.36 | 84 | 14.31 | 1.69 | 49.76 | 50.24 | 50,473 | 10 | 1.98 | 2 |
| NSW | Wollongong | Metropolitan Areas | 240.08 | 81.94 | 15.44 | 2.62 | 50.04 | 49.96 | 139,323 | 27 | 1.94 | 2 |
| NSW | Wyong | Metropolitan Areas | 700.55 | 78.53 | 18.73 | 2.74 | 51.27 | 48.73 | 173,145 | 45 | 2.60 | 4 |
| NSW | Young - Yass | Metropolitan Areas | 12,136.17 | 78.45 | 19.11 | 2.44 | 50.22 | 49.78 | 38,753 | 10 | 2.58 | 4 |
| NT | Alice Springs | Remote Communities | 569,566.00 | 91.18 | 8.2 | 0.62 | 51.1 | 48.9 | 41,962 | 5 | 1.19 | 1 |
| NT | Barkly | Very Remote Communities | 303,213.00 | 91.91 | 7.81 | 0.28 | 49.23 | 50.77 | 6,067 | 1 | 1.65 | 1 |
| NT | Daly - Tiwi - West Arnhem^*^ | Remote Communities | 112,285.21 | 90.77 | 8.97 | 0.26 | 49.4 | 50.6 | 18,374 | 0 | 0.00 | 1 |
| NT | Darwin City | Regional Centres | 40.4 | 89.26 | 9.95 | 0.79 | 48.4 | 51.6 | 29,076 | 8 | 2.75 | 4 |
| NT | Darwin Suburbs | Regional Centres | 132.44 | 87.87 | 11.19 | 0.94 | 49.81 | 50.19 | 58,532 | 12 | 2.05 | 2 |
| NT | East Arnhem | Very Remote Communities | 33,603.84 | 95.37 | 4.56 | 0.07 | 50.12 | 49.88 | 14,724 | 2 | 1.36 | 1 |
| NT | Katherine | Remote Communities | 326,256.38 | 91.93 | 7.66 | 0.41 | 49.16 | 50.84 | 21,453 | 5 | 2.33 | 3 |
| NT | Litchfield | Regional Centres | 2,938.32 | 86.67 | 12.72 | 0.61 | 44.93 | 55.07 | 23,449 | 6 | 2.56 | 4 |
| NT | Palmerston | Regional Centres | 52.53 | 93.09 | 6.5 | 0.41 | 49.77 | 50.23 | 41,432 | 9 | 2.17 | 3 |
| QLD | Bald Hills - Everton Park | Metropolitan Areas | 35.93 | 84.32 | 13.34 | 2.34 | 50.95 | 49.05 | 49,958 | 8 | 1.60 | 1 |
| QLD | Beaudesert | Medium Rural Towns | 1,628.01 | 76.63 | 20.88 | 2.49 | 49.94 | 50.06 | 16,114 | 4 | 2.48 | 4 |
| QLD | Beenleigh | Metropolitan Areas | 60.15 | 84.72 | 13.49 | 1.79 | 50.11 | 49.89 | 52,458 | 12 | 2.29 | 3 |
| QLD | Biloela | Medium Rural Towns | 28,550.18 | 83.33 | 14.62 | 2.05 | 48.5 | 51.5 | 15,053 | 5 | 3.32 | 5 |
| QLD | Bowen Basin - North | Medium Rural Towns | 79,852.28 | 87.51 | 11.52 | 0.97 | 45.61 | 54.39 | 38,247 | 8 | 2.09 | 2 |
| QLD | Bribie - Beachmere | Metropolitan Areas | 255.43 | 58.17 | 37.54 | 4.29 | 51.59 | 48.41 | 39,789 | 9 | 2.26 | 3 |
| QLD | Brisbane Inner | Metropolitan Areas | 13.56 | 90.54 | 8.42 | 1.04 | 48.99 | 51.01 | 104,824 | 39 | 3.72 | 5 |
| QLD | Brisbane Inner - East | Metropolitan Areas | 15.55 | 89.89 | 8.91 | 1.2 | 50.65 | 49.35 | 48,286 | 5 | 1.04 | 1 |
| QLD | Brisbane Inner - North | Metropolitan Areas | 30.93 | 88.99 | 9.58 | 1.43 | 50.33 | 49.67 | 111,024 | 18 | 1.62 | 1 |
| QLD | Brisbane Inner - West | Metropolitan Areas | 21.64 | 89.32 | 9.4 | 1.28 | 50.38 | 49.62 | 65,676 | 14 | 2.13 | 3 |
| QLD | Broadbeach - Burleigh | Metropolitan Areas | 33.67 | 80.77 | 16.7 | 2.53 | 51.89 | 48.11 | 72,394 | 23 | 3.18 | 5 |
| QLD | Browns Plains | Metropolitan Areas | 169.58 | 88.99 | 10.1 | 0.91 | 50.56 | 49.44 | 111,625 | 19 | 1.70 | 1 |
| QLD | Buderim | Metropolitan Areas | 76.38 | 79.25 | 17.65 | 3.1 | 52.59 | 47.41 | 62,952 | 11 | 1.75 | 2 |
| QLD | Bundaberg | Regional Centres | 3,786.01 | 73.4 | 23.59 | 3.01 | 50.93 | 49.07 | 100,388 | 23 | 2.29 | 3 |
| QLD | Burnett | Medium Rural Towns | 30,736.83 | 72.95 | 24.54 | 2.51 | 49.43 | 50.57 | 52,275 | 18 | 3.44 | 5 |
| QLD | Caboolture | Metropolitan Areas | 314.72 | 84.62 | 13.82 | 1.56 | 51.18 | 48.82 | 91,946 | 15 | 1.63 | 1 |
| QLD | Caboolture Hinterland | Regional Centres | 3,388.28 | 80.75 | 17.8 | 1.45 | 44.62 | 55.38 | 16,536 | 3 | 1.81 | 2 |
| QLD | Cairns - North | Regional Centres | 160.55 | 83.38 | 15.33 | 1.29 | 51 | 49 | 62,644 | 10 | 1.60 | 1 |
| QLD | Cairns - South | Regional Centres | 633.47 | 84.22 | 14.13 | 1.65 | 50.48 | 49.52 | 111,695 | 22 | 1.97 | 2 |
| QLD | Caloundra | Metropolitan Areas | 113.11 | 76.97 | 19.99 | 3.04 | 52.32 | 47.68 | 106,532 | 23 | 2.16 | 3 |
| QLD | Capalaba | Metropolitan Areas | 89.29 | 81.24 | 16.9 | 1.86 | 50.67 | 49.33 | 76,556 | 19 | 2.48 | 4 |
| QLD | Carindale | Metropolitan Areas | 26.51 | 85.12 | 12.95 | 1.93 | 51.41 | 48.59 | 58,034 | 10 | 1.72 | 1 |
| QLD | Centenary | Metropolitan Areas | 16.98 | 81.09 | 16.85 | 2.06 | 51.2 | 48.8 | 33,928 | 7 | 2.06 | 2 |
| QLD | Central Highlands (Qld) | Medium Rural Towns | 60,225.36 | 89.41 | 9.79 | 0.8 | 47.8 | 52.2 | 30,434 | 9 | 2.96 | 5 |
| QLD | Charters Towers - Ayr - Ingham | Medium Rural Towns | 76,209.72 | 75.67 | 21.2 | 3.13 | 49.35 | 50.65 | 42,642 | 16 | 3.75 | 5 |
| QLD | Chermside | Metropolitan Areas | 32.24 | 84.56 | 12.46 | 2.98 | 51.05 | 48.95 | 79,106 | 21 | 2.65 | 4 |
| QLD | Cleveland - Stradbroke | Metropolitan Areas | 480.9 | 76.35 | 20.91 | 2.74 | 51.03 | 48.97 | 101,572 | 20 | 1.97 | 2 |
| QLD | Coolangatta | Metropolitan Areas | 34.97 | 80.51 | 16.9 | 2.59 | 51.43 | 48.57 | 61,846 | 15 | 2.43 | 4 |
| QLD | Darling Downs - East | Regional Centres | 141,252.09 | 78.65 | 18.89 | 2.46 | 49.85 | 50.15 | 44,607 | 18 | 2.91 | 5 |
| QLD | Darling Downs (West) - Maranoa | Medium Rural Towns | 16,653.30 | 81.71 | 16.17 | 2.12 | 49.12 | 50.88 | 45,687 | 13 | 3.94 | 5 |
| QLD | Far North | Small Rural Towns | 250,877.41 | 88.41 | 10.92 | 0.67 | 47.47 | 52.53 | 33,703 | 5 | 1.48 | 1 |
| QLD | Forest Lake - Oxley | Metropolitan Areas | 63.71 | 87.55 | 10.92 | 1.53 | 48.59 | 51.41 | 85,221 | 15 | 1.76 | 2 |
| QLD | Gladstone | Large Rural Towns | 10,484.29 | 84.78 | 14.15 | 1.07 | 49.47 | 50.53 | 68,065 | 13 | 1.91 | 2 |
| QLD | Gold Coast - North | Metropolitan Areas | 40.67 | 73.82 | 23.01 | 3.17 | 52.36 | 47.64 | 73,375 | 11 | 1.50 | 1 |
| QLD | Gold Coast Hinterland | Metropolitan Areas | 827.96 | 78.42 | 19.6 | 1.98 | 50.29 | 49.71 | 21,266 | 4 | 1.88 | 2 |
| QLD | Granite Belt | Regional Centres | 8,433.97 | 72.92 | 24.28 | 2.8 | 50.44 | 49.56 | 43,323 | 10 | 2.31 | 3 |
| QLD | Gympie - Cooloola | Large Rural Towns | 6,975.22 | 73.96 | 23.63 | 2.41 | 50.38 | 49.62 | 57,340 | 17 | 2.96 | 5 |
| QLD | Hervey Bay | Regional Centres | 251.56 | 68.55 | 28.16 | 3.29 | 52.06 | 47.94 | 69,662 | 18 | 2.58 | 4 |
| QLD | Holland Park - Yeronga | Metropolitan Areas | 27.57 | 88.73 | 9.58 | 1.69 | 50.22 | 49.78 | 85,824 | 18 | 2.10 | 3 |
| QLD | Innisfail - Cassowary Coast | Regional Centres | 5,995.19 | 76.75 | 21 | 2.25 | 48.56 | 51.44 | 36,814 | 12 | 3.26 | 5 |
| QLD | Ipswich Hinterland | Metropolitan Areas | 6,135.78 | 79.69 | 18.56 | 1.75 | 49.45 | 50.55 | 73,245 | 15 | 2.05 | 2 |
| QLD | Ipswich Inner | Metropolitan Areas | 378.06 | 85.71 | 12.6 | 1.69 | 50.65 | 49.35 | 129,524 | 28 | 2.16 | 3 |
| QLD | Jimboomba | Metropolitan Areas | 582.43 | 89.27 | 10.06 | 0.67 | 50.27 | 49.73 | 75,673 | 10 | 1.32 | 1 |
| QLD | Kenmore - Brookfield - Moggill | Metropolitan Areas | 134.61 | 82.9 | 14.59 | 2.51 | 50.47 | 49.53 | 50,031 | 8 | 1.60 | 1 |
| QLD | Loganlea - Carbrook | Metropolitan Areas | 88.73 | 84.43 | 14.01 | 1.56 | 50.76 | 49.24 | 69,034 | 12 | 1.74 | 2 |
| QLD | Mackay | Regional Centres | 7,613.31 | 83.82 | 14.53 | 1.65 | 49.71 | 50.29 | 128,361 | 28 | 2.18 | 3 |
| QLD | Maroochy | Metropolitan Areas | 70.35 | 75.46 | 21.73 | 2.81 | 51.78 | 48.22 | 67,971 | 22 | 3.24 | 5 |
| QLD | Maryborough | Regional Centres | 6,752.95 | 69.82 | 27.51 | 2.67 | 49.49 | 50.51 | 51,123 | 14 | 2.74 | 4 |
| QLD | Mt Gravatt | Metropolitan Areas | 79.39 | 86.57 | 11.5 | 1.93 | 50.56 | 49.44 | 86,540 | 17 | 1.96 | 2 |
| QLD | Mudgeeraba - Tallebudgera | Metropolitan Areas | 189.2 | 85.32 | 13.25 | 1.43 | 51.54 | 48.46 | 38,104 | 4 | 1.05 | 1 |
| QLD | Nambour | Metropolitan Areas | 345.88 | 80.87 | 17.03 | 2.1 | 50.85 | 49.15 | 54,392 | 9 | 1.65 | 1 |
| QLD | Narangba - Burpengary | Metropolitan Areas | 172.41 | 84.07 | 14.41 | 1.52 | 50.84 | 49.16 | 76,186 | 7 | 0.92 | 1 |
| QLD | Nathan | Metropolitan Areas | 24.48 | 87.44 | 10.61 | 1.95 | 49.51 | 50.49 | 43,252 | 6 | 1.39 | 1 |
| QLD | Nerang | Metropolitan Areas | 123.15 | 82.95 | 14.96 | 2.09 | 50.47 | 49.53 | 73,356 | 15 | 2.04 | 2 |
| QLD | Noosa | Metropolitan Areas | 92.1 | 73.37 | 23.36 | 3.27 | 51.72 | 48.28 | 49,262 | 15 | 3.04 | 5 |
| QLD | Noosa Hinterland | Metropolitan Areas | 820.07 | 74.96 | 22.79 | 2.25 | 50.5 | 49.5 | 25,972 | 3 | 1.16 | 1 |
| QLD | North Lakes | Metropolitan Areas | 66.32 | 89.25 | 9.56 | 1.19 | 51.07 | 48.93 | 97,949 | 19 | 1.94 | 2 |
| QLD | Nundah | Metropolitan Areas | 87 | 86.94 | 11.35 | 1.71 | 49.57 | 50.43 | 46,663 | 8 | 1.71 | 1 |
| QLD | Ormeau - Oxenford | Metropolitan Areas | 519.85 | 87.69 | 11.26 | 1.05 | 50.33 | 49.67 | 180,138 | 29 | 1.61 | 1 |
| QLD | Outback - North | Remote Communities | 307,081.53 | 89.99 | 9.26 | 0.75 | 47.8 | 52.2 | 31,095 | 11 | 3.54 | 5 |
| QLD | Outback - South | Very Remote Communities | 625,221.88 | 79.68 | 18.14 | 2.18 | 50.41 | 49.59 | 17,629 | 10 | 5.67 | 5 |
| QLD | Port Douglas - Daintree | Small Rural Towns | 2,421.09 | 78.22 | 20.04 | 1.74 | 49.28 | 50.72 | 12,974 | 5 | 3.85 | 5 |
| QLD | Redcliffe | Metropolitan Areas | 213.55 | 73.7 | 23.02 | 3.28 | 52.22 | 47.78 | 68,075 | 16 | 2.35 | 3 |
| QLD | Robina | Metropolitan Areas | 33.63 | 82.19 | 15.27 | 2.54 | 52.49 | 47.51 | 57,708 | 10 | 1.73 | 2 |
| QLD | Rockhampton | Regional Centres | 18,328.13 | 81.46 | 16.47 | 2.07 | 49.98 | 50.02 | 128,183 | 26 | 2.03 | 2 |
| QLD | Rocklea - Acacia Ridge | Metropolitan Areas | 85.24 | 88.94 | 9.94 | 1.12 | 50.31 | 49.69 | 77,485 | 11 | 1.42 | 1 |
| QLD | Sandgate | Metropolitan Areas | 31.75 | 83.03 | 14.74 | 2.23 | 51.51 | 48.49 | 64,927 | 11 | 1.69 | 1 |
| QLD | Sherwood - Indooroopilly | Metropolitan Areas | 21.31 | 86.77 | 11.46 | 1.77 | 51.01 | 48.99 | 60,753 | 13 | 2.14 | 3 |
| QLD | Southport | Metropolitan Areas | 35.04 | 82.08 | 15.2 | 2.72 | 50.97 | 49.03 | 68,651 | 21 | 3.06 | 5 |
| QLD | Springfield - Redbank | Metropolitan Areas | 103.59 | 91.86 | 7.5 | 0.64 | 49.99 | 50.01 | 120,789 | 14 | 1.16 | 1 |
| QLD | Springwood - Kingston | Metropolitan Areas | 57.24 | 85.69 | 12.97 | 1.34 | 50.18 | 49.82 | 83,549 | 21 | 2.51 | 4 |
| QLD | Strathpine | Metropolitan Areas | 38.4 | 85.34 | 13.26 | 1.4 | 50.35 | 49.65 | 42,951 | 13 | 3.03 | 5 |
| QLD | Sunnybank | Metropolitan Areas | 22.16 | 85.68 | 12.72 | 1.6 | 49.69 | 50.31 | 54,686 | 8 | 1.46 | 1 |
| QLD | Sunshine Coast Hinterland | Metropolitan Areas | 1,568.02 | 78.85 | 19.22 | 1.93 | 51.09 | 48.91 | 67,521 | 10 | 1.48 | 1 |
| QLD | Surfers Paradise | Metropolitan Areas | 19.74 | 77.51 | 20.22 | 2.27 | 49.95 | 50.05 | 51,179 | 13 | 2.54 | 4 |
| QLD | Tablelands (East) - Kuranda | Regional Centres | 12,127.89 | 74.58 | 22.81 | 2.61 | 50.21 | 49.79 | 45,272 | 13 | 2.87 | 4 |
| QLD | The Gap - Enoggera | Metropolitan Areas | 96.77 | 85.68 | 12.44 | 1.88 | 50.47 | 49.53 | 57,852 | 8 | 1.38 | 1 |
| QLD | The Hills District | Metropolitan Areas | 668.68 | 84.89 | 13.8 | 1.31 | 50.3 | 49.7 | 95,502 | 19 | 1.99 | 2 |
| QLD | Toowoomba | Regional Centres | 2,258.79 | 81.05 | 16.5 | 2.45 | 51.65 | 48.35 | 173,649 | 44 | 2.53 | 4 |
| QLD | Townsville | Regional Centres | 3,826.24 | 85.07 | 13.46 | 1.47 | 49.79 | 50.21 | 204,592 | 51 | 2.49 | 4 |
| QLD | Whitsunday | Medium Rural Towns | 2,674.03 | 84.45 | 14.51 | 1.04 | 48.44 | 51.56 | 25,694 | 7 | 2.72 | 4 |
| QLD | Wynnum - Manly | Metropolitan Areas | 82.49 | 84.4 | 13.65 | 1.95 | 50.79 | 49.21 | 76,920 | 15 | 1.95 | 2 |
| SA | Adelaide City^*^ | Metropolitan Areas | 15.57 | 85.03 | 13.06 | 1.91 | 48.7 | 51.3 | 29,118 | 25 | 8.59 | 5 |
| SA | Adelaide Hills | Metropolitan Areas | 1,388.12 | 80.54 | 17.53 | 1.93 | 51.19 | 48.81 | 85,575 | 20 | 2.34 | 3 |
| SA | Barossa | Regional Centres | 2,837.15 | 77.91 | 19.72 | 2.37 | 49.45 | 50.55 | 40,361 | 9 | 2.23 | 3 |
| SA | Burnside | Metropolitan Areas | 27.52 | 76.64 | 19.72 | 3.64 | 52 | 48 | 47,810 | 12 | 2.51 | 4 |
| SA | Campbelltown (SA) | Metropolitan Areas | 25.37 | 80.24 | 16.13 | 3.63 | 51.44 | 48.56 | 58,400 | 10 | 1.71 | 1 |
| SA | Charles Sturt | Metropolitan Areas | 53.32 | 80.29 | 16.7 | 3.01 | 51.34 | 48.66 | 125,592 | 30 | 2.39 | 3 |
| SA | Eyre Peninsula and South West | Large Rural Towns | 235,368.78 | 78.36 | 19.18 | 2.46 | 48.83 | 51.17 | 59,406 | 18 | 3.03 | 5 |
| SA | Fleurieu - Kangaroo Island | Regional Centres | 7,363.57 | 63.85 | 32.11 | 4.04 | 50.64 | 49.36 | 59,364 | 17 | 2.86 | 4 |
| SA | Gawler - Two Wells | Metropolitan Areas | 306.87 | 81.46 | 15.95 | 2.59 | 50.85 | 49.15 | 41,741 | 9 | 2.16 | 3 |
| SA | Holdfast Bay | Metropolitan Areas | 13.14 | 72.09 | 23.88 | 4.03 | 52.07 | 47.93 | 37,301 | 13 | 3.49 | 5 |
| SA | Limestone Coast | Large Rural Towns | 21,337.10 | 77.2 | 19.99 | 2.81 | 48.74 | 51.26 | 69,336 | 18 | 2.60 | 4 |
| SA | Lower North^*^ | Small Rural Towns | 13,728.96 | 72.89 | 24.37 | 2.74 | 48.97 | 51.03 | 23,244 | 11 | 4.73 | 5 |
| SA | Marion | Metropolitan Areas | 56.2 | 81.12 | 16.22 | 2.66 | 51.85 | 48.15 | 101,525 | 22 | 2.17 | 3 |
| SA | Mid North | Medium Rural Towns | 14,409.49 | 73.76 | 23.23 | 3.01 | 49.32 | 50.68 | 27,705 | 11 | 3.97 | 5 |
| SA | Mitcham | Metropolitan Areas | 74.37 | 79.26 | 17.55 | 3.19 | 51.56 | 48.44 | 69,086 | 14 | 2.03 | 2 |
| SA | Murray and Mallee | Large Rural Towns | 17,851.04 | 74.31 | 22.71 | 2.98 | 48.13 | 51.87 | 74,432 | 25 | 3.36 | 5 |
| SA | Norwood - Payneham - St Peters | Metropolitan Areas | 15.1 | 79.49 | 16.85 | 3.66 | 51.37 | 48.63 | 40,062 | 15 | 3.74 | 5 |
| SA | Onkaparinga | Metropolitan Areas | 519.33 | 79.59 | 18.14 | 2.27 | 51.2 | 48.8 | 184,219 | 38 | 2.06 | 2 |
| SA | Outback - North and East | Medium Rural Towns | 642,454.00 | 84.17 | 14.32 | 1.51 | 46.7 | 53.3 | 26,540 | 7 | 2.64 | 4 |
| SA | Playford | Metropolitan Areas | 356.8 | 87.28 | 11.14 | 1.58 | 50.62 | 49.38 | 111,850 | 25 | 2.24 | 3 |
| SA | Port Adelaide - East | Metropolitan Areas | 34.84 | 86.2 | 11.79 | 2.01 | 49.49 | 50.51 | 81,940 | 17 | 2.07 | 2 |
| SA | Port Adelaide - West | Metropolitan Areas | 67.73 | 81.41 | 16.38 | 2.21 | 51.28 | 48.72 | 64,699 | 17 | 2.63 | 4 |
| SA | Prospect - Walkerville | Metropolitan Areas | 11.32 | 82.09 | 15.32 | 2.59 | 50.81 | 49.19 | 31,871 | 7 | 2.20 | 3 |
| SA | Salisbury | Metropolitan Areas | 150.17 | 84.31 | 13.79 | 1.9 | 49.92 | 50.08 | 149,090 | 30 | 2.01 | 2 |
| SA | Tea Tree Gully | Metropolitan Areas | 91.31 | 79.1 | 18.2 | 2.7 | 51.04 | 48.96 | 99,613 | 22 | 2.21 | 3 |
| SA | Unley | Metropolitan Areas | 14.27 | 77.89 | 18.56 | 3.55 | 52.7 | 47.3 | 40,327 | 10 | 2.48 | 4 |
| SA | West Torrens | Metropolitan Areas | 38.4 | 82.59 | 14.02 | 3.39 | 51.27 | 48.73 | 69,344 | 18 | 2.60 | 4 |
| SA | Yorke Peninsula | Small Rural Towns | 6,740.76 | 64.76 | 31.43 | 3.81 | 48.9 | 51.1 | 28,460 | 11 | 3.87 | 5 |
| TAS | Brighton | Regional Centres | 176.08 | 84.87 | 14.18 | 0.95 | 51.02 | 48.98 | 20,864 | 3 | 1.44 | 1 |
| TAS | Burnie - Ulverstone | Large Rural Towns | 1,604.47 | 75.97 | 21.12 | 2.91 | 51.25 | 48.75 | 51,412 | 15 | 2.92 | 5 |
| TAS | Central Highlands (Tas.) | Regional Centres | 18,426.70 | 78.16 | 20.27 | 1.57 | 46.51 | 53.49 | 12,953 | 2 | 1.54 | 1 |
| TAS | Devonport | Large Rural Towns | 1,894.35 | 75.15 | 22.18 | 2.67 | 50.41 | 49.59 | 50,338 | 10 | 1.99 | 2 |
| TAS | Hobart - North East | Regional Centres | 265.94 | 78.75 | 18.36 | 2.89 | 51.29 | 48.71 | 61,547 | 17 | 2.76 | 4 |
| TAS | Hobart - North West | Regional Centres | 168.33 | 81.83 | 15.68 | 2.49 | 50.92 | 49.08 | 58,678 | 13 | 2.22 | 3 |
| TAS | Hobart - South and West | Regional Centres | 328.51 | 79.51 | 18.25 | 2.24 | 51.89 | 48.11 | 37,982 | 8 | 2.11 | 3 |
| TAS | Hobart Inner^*^ | Regional Centres | 62.23 | 81.44 | 16.19 | 2.37 | 51.3 | 48.7 | 56,085 | 24 | 4.28 | 5 |
| TAS | Huon - Bruny Island | Regional Centres | 2,192.68 | 75.1 | 22.71 | 2.19 | 48.98 | 51.02 | 23,346 | 7 | 3.00 | 5 |
| TAS | Launceston | Regional Centres | 260.71 | 80.14 | 17.19 | 2.67 | 50.85 | 49.15 | 89,399 | 25 | 2.80 | 4 |
| TAS | Meander Valley - West Tamar | Regional Centres | 3,978.89 | 72.96 | 24.67 | 2.37 | 49.29 | 50.71 | 25,440 | 5 | 1.97 | 2 |
| TAS | North East | Regional Centres | 15,735.65 | 72.96 | 24.46 | 2.58 | 49.06 | 50.94 | 41,410 | 15 | 3.62 | 5 |
| TAS | Sorell - Dodges Ferry | Regional Centres | 694.33 | 78.31 | 19.68 | 2.01 | 50.82 | 49.18 | 19,774 | 4 | 2.02 | 2 |
| TAS | South East Coast^*^ | Small Rural Towns | 3,202.64 | 63.72 | 33.21 | 3.07 | 47.54 | 52.46 | 7,985 | 4 | 5.01 | 5 |
| TAS | West Coast^*^ | Large Rural Towns | 19,026.10 | 77.79 | 20.19 | 2.02 | 47.38 | 52.62 | 18,283 | 9 | 4.92 | 5 |
| VIC | Ballarat | Regional Centres | 448.35 | 81.19 | 16.44 | 2.37 | 51.51 | 48.49 | 123,562 | 23 | 1.86 | 2 |
| VIC | Banyule | Metropolitan Areas | 62.62 | 81.28 | 16.14 | 2.58 | 51.23 | 48.77 | 131,931 | 34 | 2.58 | 4 |
| VIC | Barwon - West | Regional Centres | 2,579.84 | 83.78 | 14.85 | 1.37 | 48.74 | 51.26 | 24,016 | 7 | 2.91 | 5 |
| VIC | Baw Baw | Large Rural Towns | 3,933.84 | 78.1 | 19.4 | 2.5 | 51.24 | 48.76 | 62,040 | 10 | 1.61 | 1 |
| VIC | Bayside | Metropolitan Areas | 37.08 | 78.5 | 18.23 | 3.27 | 52.04 | 47.96 | 106,118 | 25 | 2.36 | 3 |
| VIC | Bendigo | Regional Centres | 287.44 | 79.94 | 17.41 | 2.65 | 51.98 | 48.02 | 106,022 | 24 | 2.26 | 3 |
| VIC | Boroondara | Metropolitan Areas | 60.02 | 81.97 | 15.21 | 2.82 | 51.94 | 48.06 | 178,008 | 47 | 2.64 | 4 |
| VIC | Brimbank | Metropolitan Areas | 106.85 | 83.34 | 14.72 | 1.94 | 49.65 | 50.35 | 189,454 | 37 | 1.95 | 2 |
| VIC | Brunswick - Coburg | Metropolitan Areas | 20.41 | 88.58 | 9.45 | 1.97 | 51.66 | 48.34 | 99,537 | 26 | 2.61 | 4 |
| VIC | Campaspe | Large Rural Towns | 4,554.56 | 74.09 | 22.48 | 3.43 | 50.47 | 49.53 | 38,317 | 12 | 3.13 | 5 |
| VIC | Cardinia | Metropolitan Areas | 1,282.33 | 87.24 | 11.4 | 1.36 | 50.45 | 49.55 | 130,536 | 20 | 1.53 | 1 |
| VIC | Casey - North | Metropolitan Areas | 116.5 | 85.19 | 13.02 | 1.79 | 49.99 | 50.01 | 144,349 | 24 | 1.66 | 1 |
| VIC | Casey - South | Metropolitan Areas | 292.93 | 91.01 | 8.17 | 0.82 | 49.72 | 50.28 | 261,066 | 30 | 1.15 | 1 |
| VIC | Colac - Corangamite | Medium Rural Towns | 7,936.56 | 74.32 | 22.52 | 3.16 | 49.71 | 50.29 | 38,110 | 13 | 3.41 | 5 |
| VIC | Creswick - Daylesford - Ballan | Regional Centres | 3,666.67 | 74.68 | 23.03 | 2.29 | 49.9 | 50.1 | 31,498 | 5 | 1.59 | 1 |
| VIC | Dandenong | Metropolitan Areas | 151.05 | 83.54 | 14.19 | 2.27 | 49.24 | 50.76 | 204,942 | 42 | 2.05 | 2 |
| VIC | Darebin - North | Metropolitan Areas | 39.44 | 85.59 | 11.79 | 2.62 | 50.68 | 49.32 | 103,420 | 24 | 2.32 | 3 |
| VIC | Darebin - South | Metropolitan Areas | 14.03 | 86.85 | 10.89 | 2.26 | 52.21 | 47.79 | 56,543 | 15 | 2.65 | 4 |
| VIC | Essendon | Metropolitan Areas | 18.22 | 85.28 | 12.67 | 2.05 | 51.43 | 48.57 | 74,369 | 25 | 3.36 | 5 |
| VIC | Frankston | Metropolitan Areas | 129.6 | 83.25 | 14.64 | 2.11 | 50.86 | 49.14 | 144,615 | 32 | 2.21 | 3 |
| VIC | Geelong | Metropolitan Areas | 918.89 | 82.14 | 15.47 | 2.39 | 50.92 | 49.08 | 223,939 | 48 | 2.14 | 3 |
| VIC | Gippsland - East | Medium Rural Towns | 21,714.01 | 67.78 | 28.94 | 3.28 | 50.82 | 49.18 | 49,539 | 11 | 2.22 | 3 |
| VIC | Gippsland - South West | Medium Rural Towns | 4,381.62 | 70.26 | 26.74 | 3 | 50.67 | 49.33 | 74,753 | 20 | 2.68 | 4 |
| VIC | Glen Eira | Metropolitan Areas | 40.7 | 84.27 | 13.23 | 2.5 | 51.46 | 48.54 | 169,145 | 34 | 2.01 | 2 |
| VIC | Glenelg - Southern Grampians | Large Rural Towns | 12,946.18 | 72.7 | 23.98 | 3.32 | 50.5 | 49.5 | 36,591 | 11 | 3.01 | 5 |
| VIC | Grampians | Large Rural Towns | 38,139.60 | 74.24 | 22.24 | 3.52 | 49.34 | 50.66 | 59,413 | 21 | 3.53 | 5 |
| VIC | Heathcote - Castlemaine - Kyneton | Regional Centres | 3,920.94 | 73.91 | 23.38 | 2.71 | 49.97 | 50.03 | 54,077 | 11 | 2.03 | 2 |
| VIC | Hobsons Bay | Metropolitan Areas | 62.09 | 83.33 | 14.28 | 2.39 | 51.22 | 48.78 | 90,765 | 18 | 1.98 | 2 |
| VIC | Keilor | Metropolitan Areas | 42.43 | 79.01 | 17.3 | 3.69 | 51.22 | 48.78 | 64,063 | 12 | 1.87 | 2 |
| VIC | Kingston | Metropolitan Areas | 69.86 | 81.83 | 15.42 | 2.75 | 51.87 | 48.13 | 128,877 | 32 | 2.48 | 4 |
| VIC | Knox | Metropolitan Areas | 118.3 | 81.59 | 16.03 | 2.38 | 50.86 | 49.14 | 163,339 | 33 | 2.02 | 2 |
| VIC | Latrobe Valley | Large Rural Towns | 1,479.67 | 78.32 | 19.22 | 2.46 | 50.97 | 49.03 | 78,686 | 16 | 2.03 | 2 |
| VIC | Loddon - Elmore | Regional Centres | 7,633.52 | 72.82 | 24.12 | 3.06 | 48.83 | 51.17 | 12,655 | 3 | 2.37 | 3 |
| VIC | Macedon Ranges | Regional Centres | 992.1 | 82.17 | 16.35 | 1.48 | 50.46 | 49.54 | 35,115 | 7 | 1.99 | 2 |
| VIC | Manningham - East | Metropolitan Areas | 66.56 | 79.04 | 17.92 | 3.04 | 50.83 | 49.17 | 27,016 | 2 | 0.74 | 1 |
| VIC | Manningham - West | Metropolitan Areas | 48.67 | 78.12 | 17.82 | 4.06 | 51.77 | 48.23 | 104,807 | 16 | 1.53 | 1 |
| VIC | Maribyrnong | Metropolitan Areas | 31.23 | 89.38 | 9.19 | 1.43 | 49.34 | 50.66 | 94,251 | 29 | 3.08 | 5 |
| VIC | Maroondah | Metropolitan Areas | 61.19 | 82.28 | 15.23 | 2.49 | 51.39 | 48.61 | 118,885 | 22 | 1.85 | 2 |
| VIC | Maryborough - Pyrenees | Regional Centres | 6,172.46 | 71.22 | 25.79 | 2.99 | 48.77 | 51.23 | 27,400 | 6 | 2.19 | 3 |
| VIC | Melbourne City | Metropolitan Areas | 31.46 | 93.73 | 5.52 | 0.75 | 50.09 | 49.91 | 189,379 | 56 | 2.96 | 5 |
| VIC | Melton - Bacchus Marsh | Metropolitan Areas | 671.95 | 90.41 | 8.81 | 0.78 | 49.96 | 50.04 | 239,306 | 23 | 0.96 | 1 |
| VIC | Mildura | Large Rural Towns | 22,082.09 | 79.76 | 17.5 | 2.74 | 50.53 | 49.47 | 57,626 | 12 | 2.08 | 2 |
| VIC | Moira | Large Rural Towns | 4,046.37 | 71.4 | 24.96 | 3.64 | 49.78 | 50.22 | 30,836 | 8 | 2.59 | 4 |
| VIC | Monash | Metropolitan Areas | 79.47 | 83.32 | 13.61 | 3.07 | 49.77 | 50.23 | 201,180 | 48 | 2.39 | 3 |
| VIC | Moreland - North | Metropolitan Areas | 30.38 | 86.75 | 10.18 | 3.07 | 50.17 | 49.83 | 85,970 | 15 | 1.74 | 2 |
| VIC | Mornington Peninsula | Metropolitan Areas | 724.12 | 71.7 | 24.53 | 3.77 | 51.62 | 48.38 | 171,450 | 38 | 2.22 | 3 |
| VIC | Murray River - Swan Hill | Medium Rural Towns | 36,782.16 | 75.13 | 21.52 | 3.35 | 49.43 | 50.57 | 37,454 | 14 | 3.74 | 5 |
| VIC | Nillumbik - Kinglake | Metropolitan Areas | 751.77 | 81.97 | 16.51 | 1.52 | 50.22 | 49.78 | 68,092 | 12 | 1.76 | 2 |
| VIC | Port Phillip | Metropolitan Areas | 25.75 | 86.53 | 11.93 | 1.54 | 51.02 | 48.98 | 112,672 | 29 | 2.57 | 4 |
| VIC | Shepparton | Large Rural Towns | 2,333.16 | 80.17 | 17.25 | 2.58 | 50.13 | 49.87 | 69,684 | 15 | 2.15 | 3 |
| VIC | Stonnington - East | Metropolitan Areas | 13.72 | 82.38 | 14.72 | 2.9 | 52.07 | 47.93 | 45,418 | 16 | 3.52 | 5 |
| VIC | Stonnington - West | Metropolitan Areas | 11.93 | 84.76 | 12.8 | 2.44 | 52.2 | 47.8 | 68,620 | 25 | 3.64 | 5 |
| VIC | Sunbury | Metropolitan Areas | 260.68 | 85.47 | 13.03 | 1.5 | 50.86 | 49.14 | 50,000 | 11 | 2.20 | 3 |
| VIC | Surf Coast - Bellarine Peninsula | Metropolitan Areas | 929.66 | 74.75 | 22.72 | 2.53 | 51.5 | 48.5 | 101,887 | 17 | 1.67 | 1 |
| VIC | Tullamarine - Broadmeadows | Metropolitan Areas | 294.1 | 89.94 | 9.16 | 0.9 | 49.53 | 50.47 | 228,673 | 33 | 1.44 | 1 |
| VIC | Upper Goulburn Valley | Medium Rural Towns | 13,959.36 | 74.13 | 23.19 | 2.68 | 49.77 | 50.23 | 62,249 | 16 | 2.57 | 4 |
| VIC | Wangaratta - Benalla | Large Rural Towns | 6,545.02 | 73.26 | 23.27 | 3.47 | 51.37 | 48.63 | 49,047 | 13 | 2.65 | 4 |
| VIC | Warrnambool | Large Rural Towns | 5,496.18 | 77.78 | 19.56 | 2.66 | 51.06 | 48.94 | 53,856 | 12 | 2.23 | 3 |
| VIC | Wellington | Large Rural Towns | 10,044.82 | 75.46 | 22.16 | 2.38 | 49.49 | 50.51 | 46,416 | 13 | 2.80 | 4 |
| VIC | Whitehorse - East | Metropolitan Areas | 26.57 | 80.47 | 16.36 | 3.17 | 51.23 | 48.77 | 65,543 | 12 | 1.83 | 2 |
| VIC | Whitehorse - West | Metropolitan Areas | 38.05 | 83.29 | 13.66 | 3.05 | 51.04 | 48.96 | 118,702 | 22 | 1.85 | 2 |
| VIC | Whittlesea - Wallan | Metropolitan Areas | 997.44 | 87.53 | 11.1 | 1.37 | 49.99 | 50.01 | 281,619 | 40 | 1.42 | 1 |
| VIC | Wodonga - Alpine | Regional Centres | 13,501.04 | 78.17 | 19.34 | 2.49 | 50.75 | 49.25 | 78,341 | 18 | 2.30 | 3 |
| VIC | Wyndham | Metropolitan Areas | 544.1 | 92.31 | 7.04 | 0.65 | 49.17 | 50.83 | 341,877 | 38 | 1.11 | 1 |
| VIC | Yarra | Metropolitan Areas | 20.62 | 88.68 | 9.9 | 1.42 | 50.85 | 49.15 | 101,733 | 27 | 2.65 | 4 |
| VIC | Yarra Ranges | Metropolitan Areas | 1,605.94 | 81.55 | 16.55 | 1.9 | 50.45 | 49.55 | 159,320 | 26 | 1.63 | 1 |
| WA | Albany | Large Rural Towns | 37,888.05 | 76.14 | 21.04 | 2.82 | 50.05 | 49.95 | 65,998 | 19 | 2.88 | 4 |
| WA | Armadale | Metropolitan Areas | 559.51 | 88.11 | 10.73 | 1.16 | 50.11 | 49.89 | 109,218 | 13 | 1.19 | 1 |
| WA | Augusta - Margaret River - Busselton | Large Rural Towns | 3,664.32 | 77.33 | 20.25 | 2.42 | 50.83 | 49.17 | 64,288 | 14 | 2.18 | 3 |
| WA | Bayswater - Bassendean | Metropolitan Areas | 44.66 | 83.02 | 14.72 | 2.26 | 49.96 | 50.04 | 92,363 | 22 | 2.38 | 3 |
| WA | Belmont - Victoria Park | Metropolitan Areas | 60.11 | 86.99 | 11.41 | 1.6 | 48.6 | 51.4 | 85,217 | 18 | 2.11 | 3 |
| WA | Bunbury | Regional Centres | 5,449.51 | 80.21 | 17.71 | 2.08 | 50.22 | 49.78 | 116,367 | 27 | 2.32 | 3 |
| WA | Canning | Metropolitan Areas | 68.19 | 85.32 | 12.65 | 2.03 | 49.37 | 50.63 | 110,842 | 24 | 2.17 | 3 |
| WA | Cockburn | Metropolitan Areas | 147.98 | 86.44 | 11.96 | 1.6 | 50.13 | 49.87 | 132,734 | 26 | 1.96 | 2 |
| WA | Cottesloe - Claremont | Metropolitan Areas | 48.71 | 79.99 | 16.88 | 3.13 | 50.7 | 49.3 | 80,258 | 24 | 2.99 | 5 |
| WA | East Pilbara | Remote Communities | 390,838.28 | 95.78 | 4 | 0.22 | 42.15 | 57.85 | 27,851 | 6 | 2.15 | 3 |
| WA | Esperance | Remote Communities | 55,663.86 | 79.71 | 17.86 | 2.43 | 48.99 | 51.01 | 16,842 | 5 | 2.97 | 5 |
| WA | Fremantle | Metropolitan Areas | 41.18 | 80.71 | 16.76 | 2.53 | 50.61 | 49.39 | 44,872 | 14 | 3.12 | 5 |
| WA | Gascoyne | Very Remote Communities | 135,067.00 | 84.26 | 14.56 | 1.18 | 47.66 | 52.34 | 10,530 | 4 | 3.80 | 5 |
| WA | Goldfields | Large Rural Towns | 714,555.63 | 89.89 | 9.44 | 0.67 | 45.49 | 54.51 | 40,733 | 10 | 2.46 | 4 |
| WA | Gosnells | Metropolitan Areas | 127.3 | 86.4 | 12.08 | 1.52 | 49.29 | 50.71 | 141,279 | 21 | 1.49 | 1 |
| WA | Joondalup | Metropolitan Areas | 100.47 | 81.36 | 16.77 | 1.87 | 50.13 | 49.87 | 174,383 | 42 | 2.41 | 3 |
| WA | Kalamunda | Metropolitan Areas | 283.79 | 80.89 | 16.49 | 2.62 | 50.16 | 49.84 | 63,827 | 15 | 2.35 | 3 |
| WA | Kimberley | Remote Communities | 419,100.31 | 91.86 | 7.73 | 0.41 | 50.08 | 49.92 | 39,934 | 7 | 1.75 | 2 |
| WA | Kwinana | Metropolitan Areas | 120.01 | 90.52 | 8.55 | 0.93 | 48.06 | 51.94 | 54,672 | 7 | 1.28 | 1 |
| WA | Mandurah | Metropolitan Areas | 1,032.86 | 74.53 | 22.33 | 3.14 | 51.48 | 48.52 | 122,716 | 27 | 2.20 | 3 |
| WA | Manjimup | Regional Centres | 15,688.58 | 74 | 23.66 | 2.34 | 49.81 | 50.19 | 25,541 | 9 | 3.52 | 5 |
| WA | Melville | Metropolitan Areas | 53.46 | 79.13 | 17.72 | 3.15 | 51.48 | 48.52 | 116,222 | 25 | 2.15 | 3 |
| WA | Mid West | Large Rural Towns | 466,793.19 | 79.65 | 18.14 | 2.21 | 49.17 | 50.83 | 58,690 | 17 | 2.90 | 5 |
| WA | Mundaring | Metropolitan Areas | 685.43 | 79.85 | 17.9 | 2.25 | 47.84 | 52.16 | 47,861 | 9 | 1.88 | 2 |
| WA | Perth City | Metropolitan Areas | 43.3 | 86.41 | 11.78 | 1.81 | 49.22 | 50.78 | 126,934 | 52 | 4.10 | 5 |
| WA | Rockingham | Metropolitan Areas | 257.69 | 85.74 | 12.72 | 1.54 | 50.18 | 49.82 | 154,132 | 29 | 1.88 | 2 |
| WA | Serpentine - Jarrahdale | Metropolitan Areas | 904.76 | 89.67 | 9.54 | 0.79 | 49.37 | 50.63 | 38,687 | 6 | 1.55 | 1 |
| WA | South Perth | Metropolitan Areas | 20.31 | 81.17 | 16.42 | 2.41 | 50.17 | 49.83 | 47,958 | 8 | 1.67 | 1 |
| WA | Stirling | Metropolitan Areas | 99.32 | 82.99 | 14.28 | 2.73 | 50.73 | 49.27 | 229,809 | 50 | 2.18 | 3 |
| WA | Swan | Metropolitan Areas | 1,032.33 | 88.41 | 10.43 | 1.16 | 49.82 | 50.18 | 173,599 | 34 | 1.96 | 2 |
| WA | Wanneroo | Metropolitan Areas | 684.2 | 87.63 | 11.06 | 1.31 | 50.3 | 49.7 | 236,788 | 41 | 1.73 | 1 |
| WA | West Pilbara | Remote Communities | 116,063.99 | 96.33 | 3.57 | 0.1 | 44.08 | 55.92 | 32,895 | 9 | 2.74 | 4 |
| WA | Wheat Belt - North | Regional Centres | 110,694.80 | 75.89 | 21.7 | 2.41 | 48.26 | 51.74 | 60,818 | 24 | 3.95 | 5 |
| WA | Wheat Belt - South^*^ | Small Rural Towns | 48,762.98 | 76.32 | 21.2 | 2.48 | 48.65 | 51.35 | 20,220 | 9 | 4.45 | 5 |

^†^ Number of community pharmacies per 10,000 population in each Statistical Area Level 3 area

^*^ Outlier

ACT – Australian Capital Territory, CP – community pharmacy, CPD – community pharmacy density, ERP – estimated resident population, MMM – Modified Monash Model, no. – number, NSW – New South Wales, NT – Northern Territory, QLD – Queensland, SA – South Australia, SA3 – Statistical Area Level 3, TAS – Tasmania, WA – Western Australia
